# Deciphering the interplay between the genotoxic and probiotic activities of *Escherichia coli* Nissle 1917

**DOI:** 10.1371/journal.ppat.1008029

**Published:** 2019-09-23

**Authors:** Clémence Massip, Priscilla Branchu, Nadège Bossuet-Greif, Camille V. Chagneau, Déborah Gaillard, Patricia Martin, Michèle Boury, Thomas Sécher, Damien Dubois, Jean-Philippe Nougayrède, Eric Oswald

**Affiliations:** 1 IRSD, Université de Toulouse, INSERM, INRA, ENVT, UPS, Toulouse, France; 2 CHU Toulouse, Hôpital Purpan, Service de Bactériologie-Hygiène, Toulouse, France; McMaster University, CANADA

## Abstract

Although *Escherichia coli* Nissle 1917 (EcN) has been used therapeutically for over a century, the determinants of its probiotic properties remain elusive. EcN produces two siderophore-microcins (Mcc) responsible for an antagonistic activity against other Enterobacteriaceae. EcN also synthesizes the genotoxin colibactin encoded by the *pks* island. Colibactin is a virulence factor and a putative pro-carcinogenic compound. Therefore, we aimed to decouple the antagonistic activity of EcN from its genotoxic activity. We demonstrated that the *pks*-encoded ClbP, the peptidase that activates colibactin, is required for the antagonistic activity of EcN. The analysis of a series of ClbP mutants revealed that this activity is linked to the transmembrane helices of ClbP and not the periplasmic peptidase domain, indicating the transmembrane domain is involved in some aspect of Mcc biosynthesis or secretion. A single amino acid substitution in ClbP inactivates the genotoxic activity but maintains the antagonistic activity. In an *in vivo* salmonellosis model, this point mutant reduced the clinical signs and the fecal shedding of *Salmonella* similarly to the wild type strain, whereas the *clbP* deletion mutant could neither protect nor outcompete the pathogen. The ClbP-dependent antibacterial effect was also observed *in vitro* with other *E*. *coli* strains that carry both a truncated form of the Mcc gene cluster and the *pks* island. In such strains, siderophore-Mcc synthesis also required the glucosyltransferase IroB involved in salmochelin production. This interplay between colibactin, salmochelin, and siderophore-Mcc biosynthetic pathways suggests that these genomic islands were co-selected and played a role in the evolution of *E*. *coli* from phylogroup B2. This co-evolution observed in EcN illustrates the fine margin between pathogenicity and probiotic activity, and the need to address both the effectiveness and safety of probiotics. Decoupling the antagonistic from the genotoxic activity by specifically inactivating ClbP peptidase domain opens the way to the safe use of EcN.

## Author summary

*Escherichia coli* Nissle 1917 (EcN) has been used as a probiotic for over a century. However, it produces the genotoxin colibactin, which has been linked to the virulence of certain *E*. *coli* strains and could promote colorectal cancer. Administering a potentially pro-carcinogenic strain as a probiotic raises public health concerns. Therefore, our aim was to separate EcN genotoxic activity from its antagonistic effect, one of the most distinctive features of the probiotic property of EcN. We demonstrated that the microcin activity of EcN requires ClbP, an enzyme essential for colibactin production, and another enzyme involved in the synthesis of the siderophore salmochelin. This interplay was found in other *E*. *coli* strains, mainly isolated from urine. Therefore, *E*. *coli* strains utilize determinants from three distinct metabolic pathways to synthesize siderophore-microcins. Interestingly, the ClbP peptidase activity mandatory for colibactin production, has no role in siderophore-microcin activity, which provides a way to construct a non-genotoxic strain that retains its antibacterial activity. This intricate interplay between probiotic and pathogenic determinants confirms that biosynthetic pathways should be studied at the level of protein domains rather than that of whole proteins.

## Introduction

The probiotic *Escherichia coli* strain Nissle 1917 (EcN) was isolated during World War I by Alfred Nissle in a soldier who resisted a severe diarrhea outbreak [1,2]. EcN was initially studied for its ability to fight bacterial gastrointestinal infections. It was demonstrated to impede intestinal colonization by *Salmonella enterica* serovar Typhimurium [3,4] and to exhibit an antibacterial activity against enterohemorrhagic *E*. *coli* strains [5]. EcN is an excellent colonizer of the human gut, and exhibits beneficial effects in various intestinal dysfunctions such as acute diarrhea in infants and toddlers [6], chronic constipation [7], and abdominal pain in patients with irritable bowel syndrome [8]. It has been widely used in the treatment of inflammatory bowel diseases [1] and has proven to be as effective as the gold standard mesalazine for the maintenance of remission in ulcerative colitis in children and adults [9].

EcN probiotic activity is believed to be based on multiple peculiar properties and fitness determinants, including antibacterial activities against other bacteria [10]. Thanks to an extensive list of siderophores (enterobactin, salmochelin, yersiniabactin, and aerobactin) and multiple siderophore receptors and iron transport systems, EcN reduces *S*. Typhimurium intestinal colonization by competing for iron [3]. Enterobactin, salmochelin and yersiniabactin are nonribosomal peptides (NRP) or polyketide (PK)-NRP hybrids, which are synthesized by NRP synthetases and PK synthases (NRPS and PKS) activated by a cognate phosphopantetheinyl transferase (PPTase). In addition to this competition for a limiting nutrient, EcN exhibits a direct antibacterial activity linked to the production of two microcins (Mcc), H47 (MccH47) and M (MccM) [4,11–13]. Mcc are secreted low-molecular weight peptides that are synthesized by ribosomes and posttranslationally modified, and which display a potent bactericidal activity against phylogenetically-related bacteria [14,15]. MccH47 and MccM are called “siderophore-Mcc” because they are modified posttranslationally by the linkage of a catechol siderophore [13,16]. The C-terminus of the Mcc peptide is covalently bound with a linearized and glycosylated derivative of enterobactin [13,16,17]. This siderophore moiety is recognized by the catecholate-siderophore receptors of the target bacterium [12,16]. The siderophore-Mcc can therefore enter and kill the sensitive bacterium by a “Trojan Horse” stratagem, by mimicking the iron-siderophore complexes.

Comparative genomic analyses have shown that EcN is closely related to pathogenic *E*. *coli* strains such as the uropathogenic strain CFT073 [18–20]. EcN and CFT073 share eight genomic islands, including the *pks/clb* island encoding a NRPS-PKS assembly line that synthesizes the genotoxin colibactin [21,22]. Colibactin is produced as a prodrug moiety that is exported in the periplasm by the efflux pump ClbM [23] and then hydrolyzed by the periplasmic membrane-bound ClbP protein with a peptidase activity, which releases the active colibactin [24,25]. Colibactin is not only a *bona fide* virulence factor [26,27] but also a putative procarcinogenic compound. Colibactin alkylates the host cell DNA, resulting in DNA crosslinks, double-strand breaks, chromosome aberrations and gene mutations both *in vitro* and *in vivo* [21,22,28–32]. Colibactin-producing *E*. *coli* are overrepresented in biopsies of patients with colorectal cancer [33,34] and they were shown to promote colorectal cancer in mouse models [33,35].

The ambivalence between the pathogenic and probiotic potential of EcN was uncovered when we showed that certain enzymes of the *pks/clb* island enable the synthesis of analgesic lipopeptides [36] and that the probiotic properties of EcN are related to the presence of the pathogenicity island [37]. In the Olier et al. (2012) study, we inactivated the gene that encodes the phosphopantetheinyl transferase (PPTase) ClbA, which was thought to be specific for colibactin synthesis [37]. However, more recent work has shown that ClbA has a pleiotropic effect and also modulates the synthesis of siderophores as well as that of the analgesic lipopeptides [36,38]. As might be expected, use of a probiotic strain that produces a genotoxin is a public health concern. We therefore attempted to clearly decouple the genotoxic from the antagonistic activities. In this study, we used our knowledge of the biosynthetic pathway of colibactin and other secondary metabolites produced directly or indirectly by the *pks/clb* island to specifically abrogate the genotoxic activity of colibactin. We examined the ability of mutants to inhibit the growth of pathogenic bacteria while still producing beneficiary secondary metabolites. We successfully decoupled the antibacterial activity from the genotoxic activity, consequently opening the way to optimize EcN. However, we were surprised to observe that the *pks/clb* island was even more intimately connected to EcN probiotic activity than we expected, and we propose that there might be a co-evolution of pathogenic and probiotic properties in bacteria. EcN is, to some extent, like the “miracle drug” aspirin. Although, like aspirin, this bacterial strain has been used successfully for over a century, it is crucial to understand the mode of action and to take into account the safety and potential side effects.

## Results

### EcN antibacterial activity requires ClbP but not the other components of the colibactin synthesis pathway

**Fig 1:**
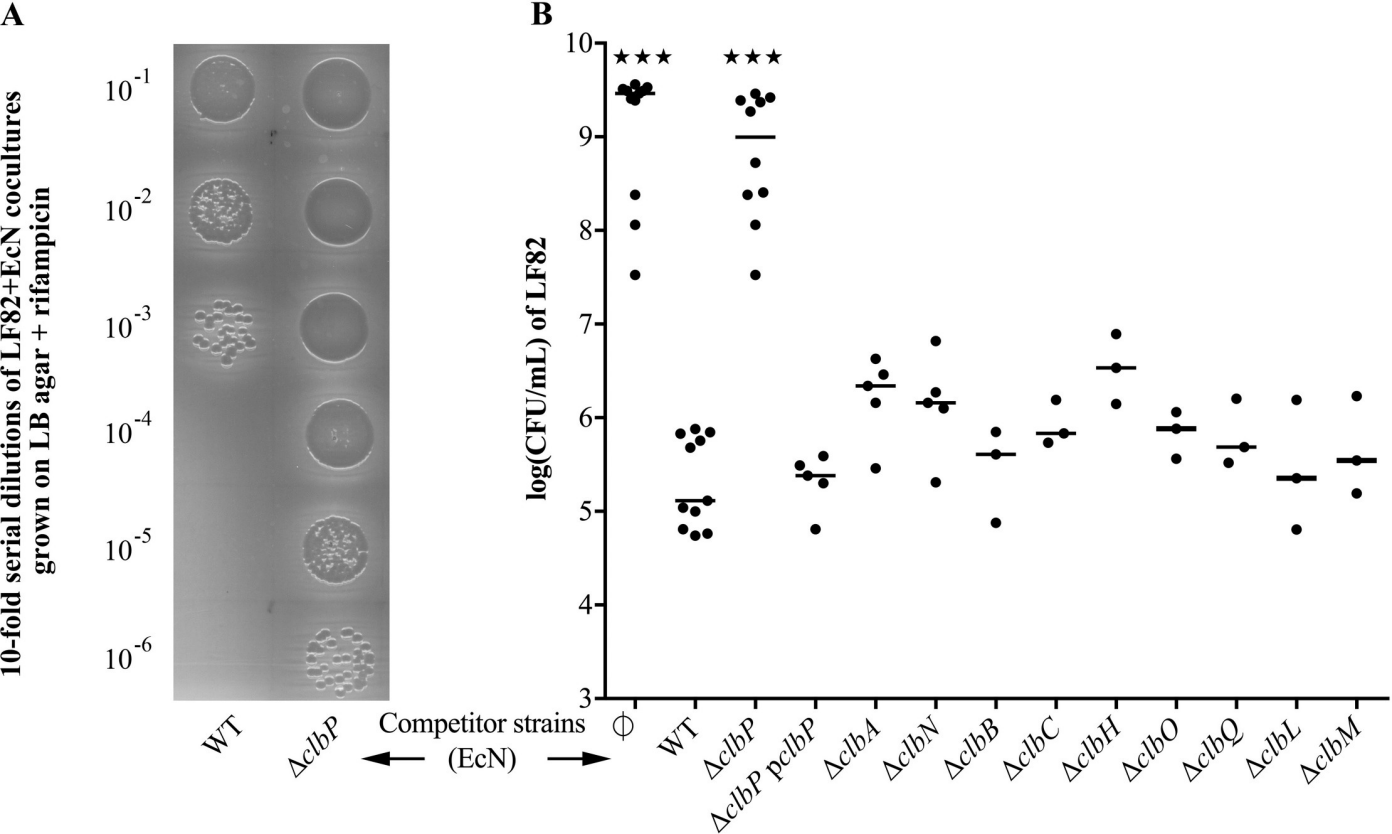
Role of the *pks*/*clb* island in EcN antibacterial activity on LF82. (**A)** Serial dilutions (ten-fold) of 24-hour co-cultures of *E*. *coli* LF82 (rifampicin resistant) with wild-type (WT) *E*. *coli* strain Nissle 1917 (EcN) or the mutant for the colibactin maturing peptidase ClbP, spotted (10 μL) on LB plate containing rifampicin and incubated overnight at 37°C. Colony forming unit (CFU) counts enable to determine LF82 growth following the 24-hour co-culture. **(B)** CFU counts of *E*. *coli* LF82 following a 24-hour co-culture in M63 medium with WT EcN, gene deletion for the phosphopantetheinyl transferase ClbA, the peptidase ClbP and the corresponding complemented mutant (pclbP), the polyketide synthases (PKS) ClbC and ClbO, the nonribosomal peptide synthases (NRPS) ClbH and ClbN, the hybrid PKS-NRPS ClbB, the putative amidase ClbL, the efflux pump ClbM, and the thioesterase ClbQ. LF82 was also cultured alone as a control (∅). The medians and individual results of independent experiments are shown. One-way ANOVA and Bonferroni post-tests in comparison with co-culture with WT; ★★★P<0.001.

In order to specifically decouple the genotoxic activity from the antagonistic activity, we tested the antibacterial activity of the EcN mutant deleted for ClbP that allows the maturation of precolibactin in genotoxic colibactin [24,25]. We compared it to the pleiotropic ClbA mutant coding for a PPTase [27,37,38]. We performed co-culture experiments with the wild-type EcN, the EcN Δ*clbA* and Δ*clbP* mutants, and the Crohn’s disease-associated *E*. *coli* strain LF82 which have been previously shown to be susceptible to EcN [39,40]. Colony forming units (CFU) counts showed that the EcN strain strongly inhibited LF82 growth. EcN antibacterial activity on LF82 was not altered in a Δ*clbA* mutant but was completely lost in a Δ*clbP* mutant (Fig 1). A kinetic experiment indicated that EcN inhibitory activity on LF82 started 6 hours post-inoculation and reached its maximum 8 hours post-inoculation, at the beginning of the stationary phase (S1 Fig). LF82 growth was not altered at any time by the Δ*clbP* mutant, further indicating that EcN antibacterial effect requires ClbP. This EcN ClbP-dependent inhibitory activity was also observed with other pathogenic strains of *E*. *coli* and closely related bacteria species, such as *Salmonella enterica* subsp. *enterica* Typhimurium (*S*. Typhimurium), and *Enterobacter aerogenes* (S2 Fig).

To further determine whether other components of the colibactin synthesis pathway besides ClbP are required for EcN antibacterial activity, the inhibitory effect of mutants for the PKS ClbC and ClbO, the NRPS ClbH and ClbN, the hybrid PKS-NRPS ClbB, the putative amidase ClbL, the efflux pump ClbM, and the thioesterase ClbQ were assessed against LF82. EcN antibacterial activity against LF82 was not altered in any of these mutants (Fig 1). These results confirm that mature colibactin or the cleavage product N-acyl-D-asparagine are not essential for EcN antibacterial activity against LF82. Therefore, the antagonistic activities of EcN are clearly associated with the presence of the *pks/clb* island and ClbP but colibactin is not involved in EcN inhibitory activity.

### EcN ClbP-dependent antibacterial activity requires MccH47 and MccM

**Fig 2:**
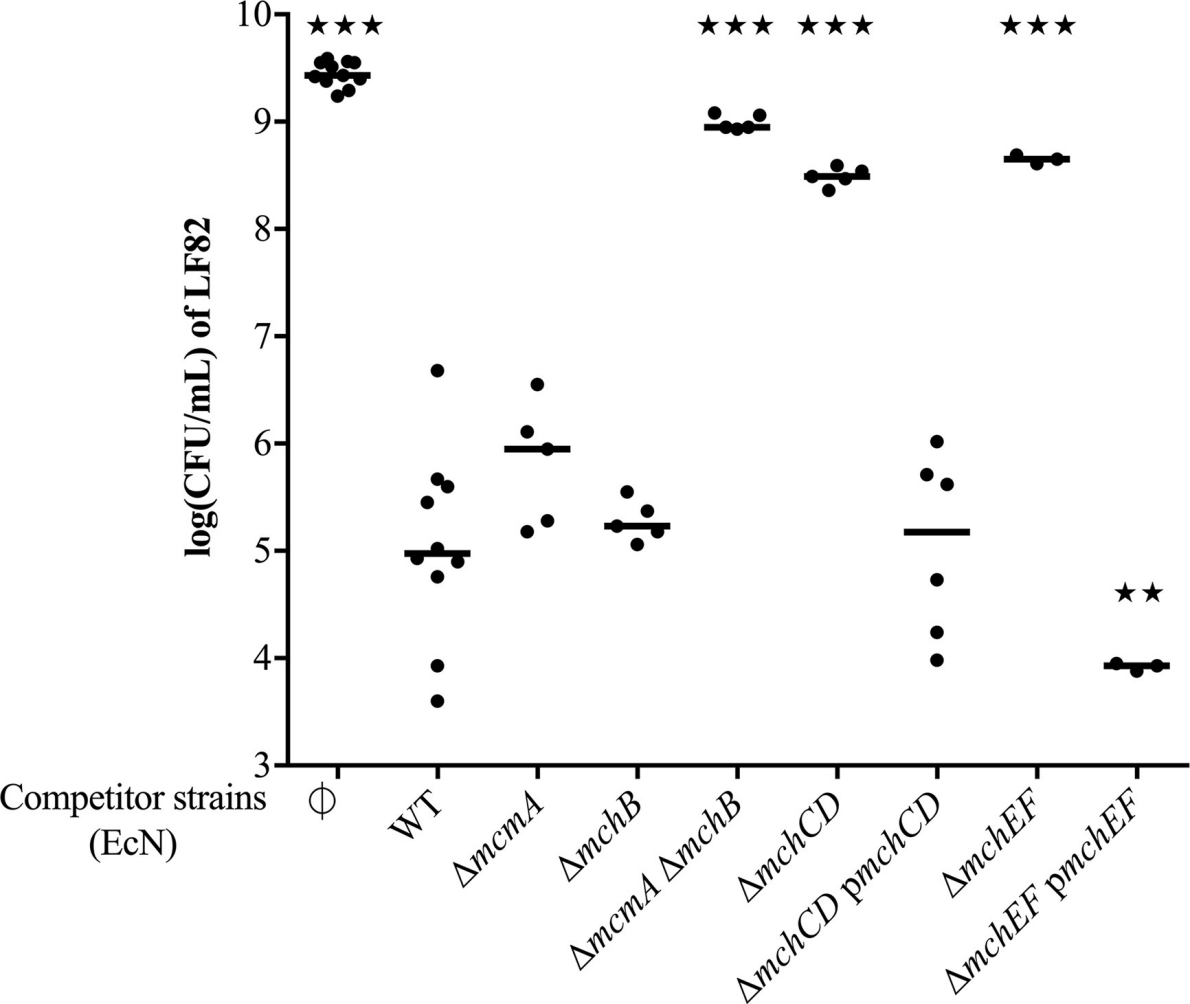
Role of the microcin gene cluster in EcN antibacterial activity on LF82. Colony forming unit (CFU) counts of *E*. *coli* LF82 following a 24-hour co-culture in M63 medium with wild-type (WT) *E*. *coli* strain Nissle 1917 (EcN), EcN mutant for microcin M (MccM) precursor gene *mcmA*, for microcin H47 (MccH47) precursor gene *mchB*, for both *mcmA mchB* genes; EcN mutants and complemented strains for *mchC mchD* genes responsible for posttranslational modifications, and for *mchE mchF* genes that encode the MccM and MccH47 efflux pump. LF82 was also cultured alone as a control (∅). The medians and individual results of independent experiments are shown. One-way ANOVA and Bonferroni post-tests in comparison with co-culture with WT; ★★P<0.01; ★★★P<0.001.

Previous studies have associated EcN antibacterial activity with MccH47 and MccM [4,11,14,15]. Therefore, we performed co-culture experiments with LF82, EcN and mutants in MccH47 and MccM production systems. EcN antibacterial activity against LF82 was not affected by the deletion of the MccM precursor gene *mcmA* alone or the MccH47 precursor gene *mchB* alone (Fig 2). In contrast, deletions of both *mcmA* and *mchB* completely abrogated the inhibitory effect of EcN on LF82. Similarly, deletion of the MccM and MccH47 efflux pump encoding genes *mchE* and *mchF* resulted in a loss of antibacterial activity (Fig 2). The trans-complementation of *mchE* and *mchF* increased EcN inhibitory activity compared to the wild-type EcN strain (Fig 2), probably because of an increase in Mcc export following overexpression of the MchE-MchF efflux pump. None of these mutations in the Mcc production system affected the ability of EcN to produce active colibactin (S3 Fig).

To further confirm the role of MccH47 and MccM in EcN antibacterial activity, plasmids that encode MccH47 or MccM immunity genes were transformed in LF82, and the resulting resistance of the strains was assessed against EcN (S4 Fig). EcN Δ*mchB* mutant antibacterial activity was almost completely abrogated on LF82 that carries the MccM immunity gene *mcmI* (S4A Fig). A similar result was obtained with the Δ*mcmA* mutant and LF82 that carries MccH47 immunity gene *mchI* (S4B Fig). Overall, these results confirmed that EcN inhibitory activity against LF82 is due to MccH47 and MccM, and that this activity is ClbP-dependent.

### EcN ClbP-dependent antibacterial activity is due to the production of siderophore-Mcc

**Fig 3:**
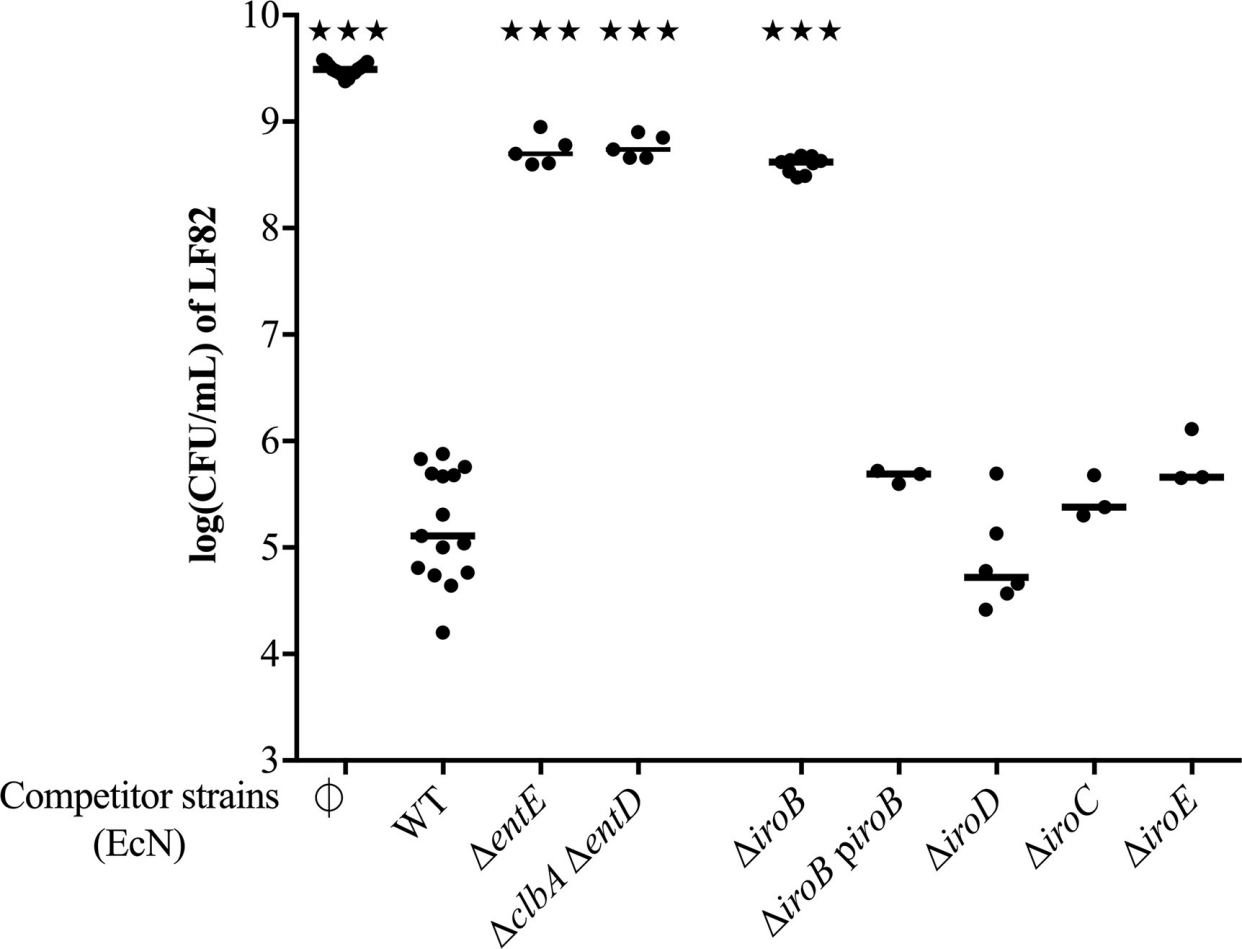
Role of siderophores in EcN antibacterial activity on LF82. Colony forming unit (CFU) counts of *E*. *coli* LF82 following a 24-hour co-culture in M63 medium with wild-type (WT) *E*. *coli* strain Nissle 1917 (EcN), EcN mutant for *entE* that encodes the enterobactin synthase E, and the double mutant for the phosphopantetheinyl transferases ClbA and EntD; EcN mutant and complemented strain for the glucosyltransferase IroB, the cytoplasmic esterase IroD, the periplasmic esterase IroE, and the export protein IroC. LF82 was also cultured alone as a control (∅). The medians and individual results of independent experiments are shown. One-way ANOVA and Bonferroni post-tests in comparison with co-culture with WT; ★★★P<0.001.

MccH47 and MccM can be modified posttranslationally by the linkage of a catechol siderophore to form a “siderophore-Mcc” [13]. Therefore, we hypothesized that the ClbP-dependent antibacterial activity might be dependent on these modified forms of Mcc. In fact, EcN antibacterial activity against LF82 was shown to be strongly reduced in a Δ*entE* mutant deprived of the enzyme 2,3-dihydroxybenzoate-AMP ligase essential for siderophore enterobactin production [41] (Fig 3). Similar results were obtained with the EcN Δ*clbA* Δ*entD* double mutant which was unable to produce enterobactin [38] (Fig 3).

The two genes responsible for enterobactin glycosylation and esterification (*mcmL* and *mcmK*) are missing from the EcN Mcc gene cluster [18,42]. As a result, whether MccH47 and MccM are siderophore-Mcc or unmodified Mcc is still being debated [13]. Considering that EcN carries the McmL and McmK homologs, the glucosyltransferase IroB and esterase IroD respectively [13], we investigated the interplay between the Mcc and the salmochelin production systems. The antibacterial activity of EcN mutants for genes that encode the glucosyltransferase IroB, the cytoplasmic esterase IroD, the periplasmic esterase IroE, and the export protein IroC [43,44] was compared to the activity of the wild-type EcN strain. Only *iroB* deletion led to a significant decrease in EcN antibacterial activity (Fig 3). Complementation of the Δ*iroB* mutant fully restored the antibacterial activity. None of these mutations in the *iroA* locus affected EcN ability to cause megalocytosis, the enlargement of infected cells indicative of colibactin genotoxic effect (S3 Fig). These results suggest that IroB could be responsible for enterobactin glycosylation, which enables the linkage of Mcc precursor proteins to the siderophore-derived moiety in the absence of McmL.

MchC and MchD are respective homologous to MceJ and MceI of *K*. *pneumoniae* strain E492 [13]. These proteins form a complex responsible for the linkage of glycosylated enterobactin derivatives to MccE492 the precursor peptide MceA [45]. The EcN mutant for *mchC* and *mchD* lost the antibacterial effect against LF82, whereas complementation restored the initial phenotype (Fig 2). These results indicate that the posttranslational modification of MccH47 and MccM with an enterobactin-derived moiety is required for EcN antibacterial activity. In short, EcN ClbP-dependent antibacterial activity is due to siderophore-Mcc.

### The ClbP transmembrane domain, but not the periplasmic peptidase domain, is required for the antibacterial activity of EcN

**Fig 4:**
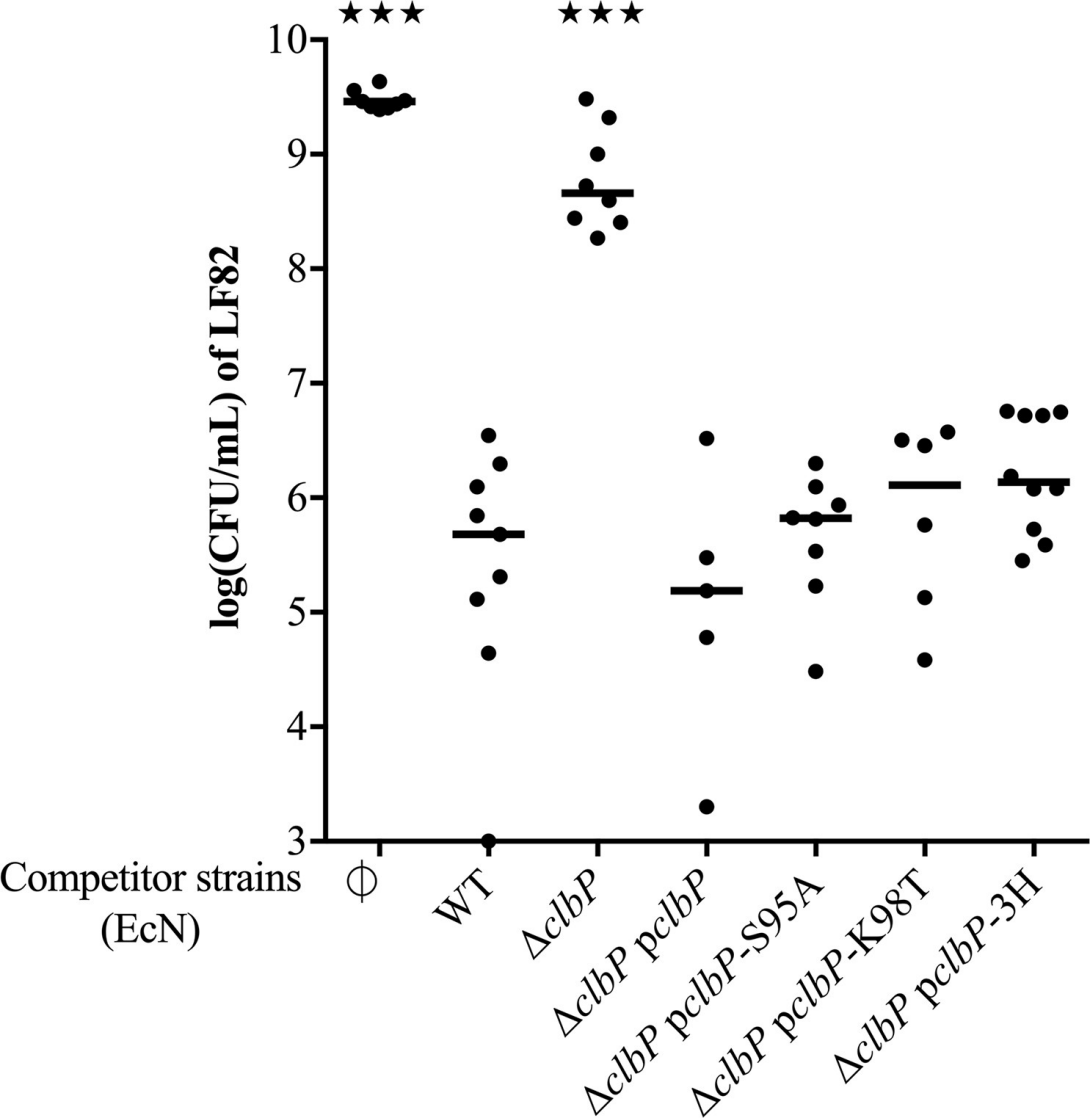
Role of ClbP catalytic and transmembrane domains in EcN antibacterial activity on LF82. Colony forming unit (CFU) counts of *E*. *coli* LF82 following a 24-hour co-culture in M63 medium with wild-type (WT) *E*. *coli* strain Nissle 1917 (EcN), the *clbP* gene deletion and complemented mutant with a plasmid that encodes wild-type ClbP (p*clbP*), plasmids that encode ClbP with a mutation S95A or K98T in the catalytic site, and a plasmid that encodes a fusion between the alkaline phosphatase PhoA and the ClbP C-terminal sequence from amino-acid 390 (p*clbP*-3H). LF82 was also cultured alone as a control (∅). Medians and individual results of independent experiments are shown. One-way ANOVA and Bonferroni post-tests in comparison with co-culture with WT; ★★★P<0.001.

To further explore the role of ClbP in siderophore-Mcc production, we examined whether ClbP catalytic activity is required for EcN antibacterial activity. S95 and K98 are key residues for ClbP peptidase activity, and mutants for these residues fail to cleave precolibactin to release mature active genotoxin [24,25]. Co-culture experiments were performed with LF82 and the EcN Δ*clbP* mutant complemented with plasmids that encode the wild-type ClbP protein, or the ClbP protein that harbors the substitutions S95A or K98T. EcN Δ*clbP* mutants complemented with ClbP S95A or K98T demonstrated antibacterial activities similar to those of the wild-type ClbP protein (Fig 4), whereas they lost their ability to cause megalocytosis linked with the colibactin genotoxic effect (S3 Fig).

To exclude a role of another putative catalytic activity in ClbP periplasmic domain, this domain was replaced by the enzymatic domain of the alkaline phosphatase PhoA, as previously reported [46]. The PhoA domain was fused with the ClbP N-terminal signal sequence which allows the translocation to periplasm, and the ClbP C-terminal sequence from amino-acid 390; the residues forming the three transmembrane helices being 390–412, 433–455, and 465–485 [24]. An EcN Δ*clbP* mutant transformed with a plasmid bearing this fusion demonstrated a similar inhibitory activity against LF82 as the EcN WT strain (Fig 4), whereas it did not cause megalocytosis (S3 Fig). Therefore, the C-terminal domain of ClbP that comprises the three transmembrane helices is essential for EcN antibacterial activity, as opposed to the ClbP periplasmic peptidase domain that is crucial only for genotoxic activity. A previous study from Dubois et al. identified ClbP orthologs, called “ClbP-like” proteins, in γ-proteobacteria and in firmicutes [24]. We transformed five of these ClbP orthologs from the following bacteria species: *Hahella chejuensis*, *Bacillus weihenstephanensis*, *B*. *mycoides*, *B*. *pseudomycoides*, and *Clostridium cellulolyticum* in the EcN Δ*clbP* mutant and tested their antibacterial activity. The ClbP-like proteins shared a particular cellular localization and topology, namely a large N-terminal extracytoplasmic peptidase domain anchored to the inner membrane by three C-terminal transmembrane helices, although they differ in their amino-acid sequences. The expression of each ortholog in a EcN Δ*clbP* background fully restored the antibacterial activity of EcN (S5 Fig). The efficient xeno-complementations support the functional promiscuity of ClbP-like proteins for both the peptidase domain [24] and the trans membrane domain. Since both the siderophore-Mcc efflux pump MchEF and the C-terminal domain of ClbP crucial for EcN antagonistic activity are inserted in the bacterial inner membrane [24,47,48], this result suggests the hypothesis that ClbP could be involved in the export of the two siderophore-Mcc, by facilitating the function of MchEF. To assess this hypothesis, we overexpressed MchEF or ClbP in a Δ*clbP* and a Δ*mchEF* mutant, respectively. The overexpression of the Mcc efflux pump restored the antagonistic activity in the EcN Δ*clbP* mutant, whereas the overexpression of ClbP had no effect on the activity of the EcN Δ*mchEF* mutant (S6 Fig). Altogether, these results support a model in which ClbP could act as a cofactor which facilitates the export of EcN siderophore-Mcc.

### An EcN strain with a point mutation in the *clbP* gene is non-genotoxic but keeps the antagonist activity, and reduces S. Typhimurium intestinal colonization and virulence

**Fig 5:**
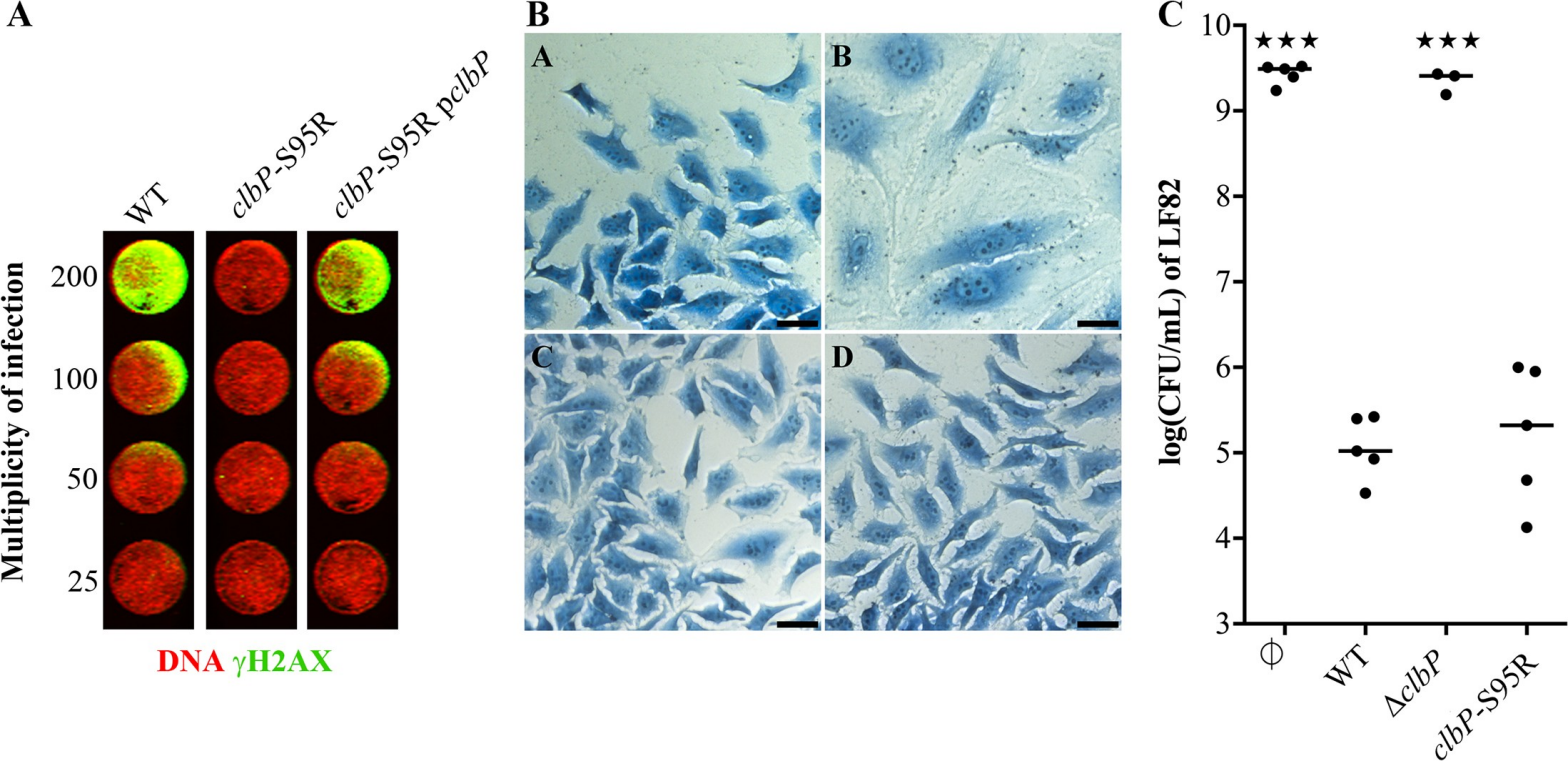
A genomic point mutation that inactivates ClbP catalytic domain abrogates EcN genotoxicity but not the antibacterial activity on LF82. **(A)** HeLa cells were transiently infected with wild-type *E*. *coli* Nissle (WT), a genome edited mutant with a single chromosomal nucleotide change in the *clbP* gene that inactivates the catalytic site (*clbP*-S95R), and the genome edited mutant complemented with the plasmid p*clbP*. Cells were then fixed, permeabilized and stained with rabbit monoclonal anti-gamma-H2AX followed by an infrared fluorescent secondary antibody (pseudocolored in green). DNA was counterstained with RedDot2. (**B)** HeLa cells were transiently infected with wild-type *E*. *coli* Nissle (photo B), a *clbP* gene deletion mutant (C), and the genome edited *clbP*-S95R mutant (D). These cells were then washed and incubated with gentamicin for 72 hours before staining with Giemsa. The control is shown in photo A. Bars represent 50 μm. **(C)** Colony forming unit (CFU) counts of *E*. *coli* LF82 following a 24-hour co-culture in M63 medium with wild-type (WT) *E*. *coli* strain Nissle 1917 (EcN), the *clbP* gene deletion mutant (ΔclbP), and the genome edited mutant with a single nucleotide change in the *clbP* gene that results in an S95R mutation in the catalytic site (*clbP*-S95R). LF82 was also cultured alone as a control (∅). Medians and individual results of independent experiments are shown. One-way ANOVA and Bonferroni post-tests in comparison with co-culture with WT; ★★★P<0.001.

As a proof of the concept that a non-genotoxic EcN strain keeping its antagonistic activity could be engineered, we performed genome editing to construct an EcN mutant strain that exhibits a point nucleotide mutation in the chromosomic *clbP* gene, which leads to an S95R mutation in the ClbP catalytic site. This mutant did not produce significant amounts of precolibactin cleavage product N-acyl-D-asparagine (S7 Fig), and was not genotoxic for infected HeLa cells as shown by the negative megalocytosis and phosphorylation of histone H2AX phenotypes (Fig 5A and 5B). The *clbP*-S95R mutant still exhibited an antibacterial activity towards LF82 similar to that of the wild-type genotoxic EcN strain (Fig 5C).

**Fig 6:**
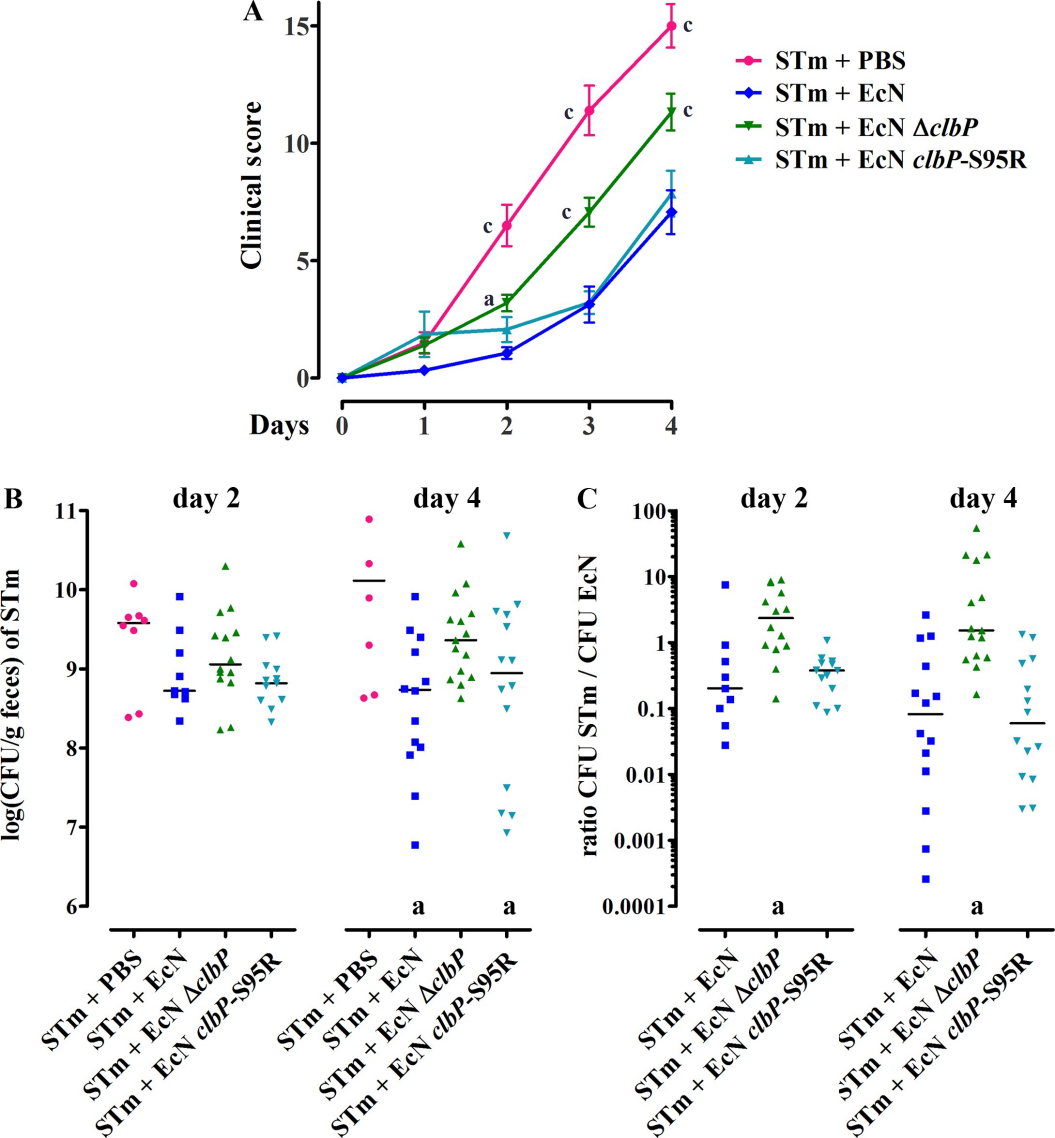
A *clbP* deletion, but not a genomic point mutation that inactivates ClbP catalytic domain, impairs EcN protection against the enteric pathogen *Salmonella* Typhimurium in mice. C57BL/6 female mice were treated with 20 mg streptomycin *per os*, then 24 h later infected orally with 10^9^ *S*. Typhimurium (STm) in PBS or co-administered with 10^9^ *S*. Typhimurium and 10^9^ EcN wild-type, Δ*clbP* or *clbP*S95R strains. **(A)** The mice were monitored for clinical signs (weight loss, diarrhea, signs of abdominal pain) daily during 4 days. Each point corresponds to the mean clinical score +/- SEM of 10 to 15 animals per group in three independent experiment. The animals were scored blindly (without knowledge of the infecting bacteria) in two of the three experiments. Two-way ANOVA with Bonferroni post-test compared to STm + EcN, a: p<0.05, c: p<0.001. **(B)** The fecal shedding of STm was examined by enumeration of the feces collected at day 2 and 4 after infection. The median and individual results are shown. One way ANOVA of log-transformed CFU counts compared to STm + PBS, a: p<0.05. **(C)** Fecal counts of STm and EcN were used to determine the competitive index (CFU STm / CFU EcN). One way ANOVA compared to STm + EcN *clbP*S95R, a: p<0.05.

The EcN probiotic is well known to offer protection against enteric pathogens such as *Salmonella*, by competing for iron and producing the siderophore-Mcc [3,4]. Thus, we next examined whether the EcN wild-type, Δ*clbP* and *clbP-*S95R mutants reduces *S*. Typhimurium intestinal colonization and pathogenesis using an *in vivo* model. We utilized C57BL/6 mice treated with streptomycin (to ensure a high colonization) then 24 h later infected with *S*. Typhimurium alone, or co-administered with *S*. Typhimurium and each EcN strain [3,4,49]. The mice were monitored for clinical signs (weight loss, diarrhea, signs of abdominal pain) and the bacterial colonization was examined by enumeration of the feces, during 4 days (the point where the experiment must be arrested because of the lethality). When administered alone, *S*. Typhimurium readily colonized the intestine and this was associated with a high clinical score linked to a strong enteric salmonellosis (Fig 6). In animals co-administered with the wild-type EcN, there was a marked reduction in the clinical scores and in *S*. Typhimurium fecal colonization (Fig 6A and 6B). By day 2 following infection, EcN significantly outcompeted *S*. Typhimurium (Fig 6C). In contrast, animals co-administered with the EcN Δ*clbP* mutant exhibited higher clinical scores and reduced antagonism of *S*. Typhimurium colonization, demonstrating the role of ClbP in EcN beneficial effect during acute *Salmonella* colitis. The EcN *clbP-*S95R strain reduced substantially the fecal shedding and outcompeted *S*. Typhimurium, and diminished the clinical scores, similarly to the wild-type EcN (Fig 6).

Altogether, these results show that it is possible to decouple the genotoxic activity of EcN from its antibacterial activity, but also that the biosynthetic pathways of colibactin and siderophore-Mcc are more entangled than we initially thought.

### The ClbP-dependent antibacterial activity is observed in a subset of *E*. *coli* strains that carry a truncated Mcc gene cluster and the *pks* island

Comparative genomic analyses have shown that EcN is closely related to *E*. *coli* pyelonephritis strain CFT073 and the asymptomatic bacteriuria strain ABU83972 [18]. These three strains, as well as the reference strain ATCC®25922, carry the *pks* island, the *iroA* locus, and a truncated Mcc gene cluster deprived of genes *mcmL/mchA* and *mcmK/mchS1*. Therefore, we assessed whether the siderophore-Mcc antibacterial effect of these strains was ClbP-dependent, as observed in EcN. The inhibitory effect of two sets of *E*. *coli* strains was tested in co-culture experiments against LF82, as well as their respective Δ*clbP* mutants: i) strains similar to EcN that carry both a truncated Mcc gene cluster and the *pks* island: strains CFT073, ABU83972, and ATCC®25922; and ii) strains that carry the *pks* island but which are deprived of Mcc encoding genes: the human commensal strain M1/5, the meningitis-causing strain SP15, the murine commensal strain NC101, and the laboratory strain MG1655 that hosts a bacterial artificial chromosome (BAC) bearing the *pks* island. The three wild-type strains that carry both a truncated Mcc gene cluster and the *pks* island exhibited a marked inhibitory effect as observed in EcN (S8 Fig). The inhibitory effect of all three corresponding Δ*clbP* mutant strains was significantly reduced, whereas ClbP complementation restored the initial phenotype (S8 Fig). In contrast, in strains carrying only the *pks* island, there was no significant difference in LF82 growth whether it was cultivated with the wild-type strains or the Δ*clbP* mutants (S8 Fig). Cumulatively, these results show that the peptidase ClbP is involved in MccH47 and MccM antibacterial activity in *E*. *coli* strains that carry both the *pks* island and a truncated form of the Mcc gene cluster. Our results also show that this association is present in both pathogenic and probiotic strains.

### Distribution of pks, salmochelin and the MccH47 and MccM gene clusters in an *E*. *coli* population

**Fig 7:**
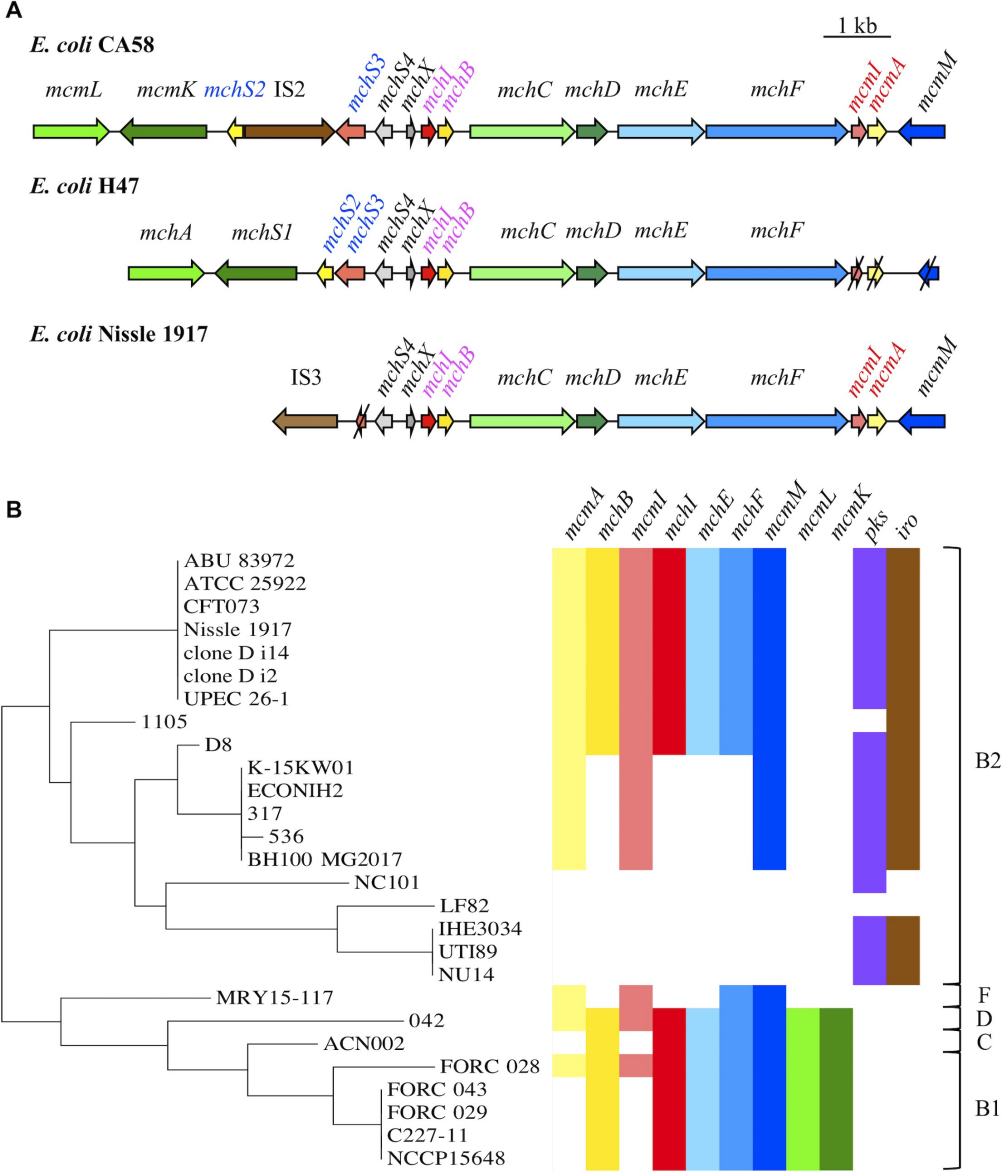
Organization and distribution of microcin, *pks* and *iro* island in *E*. *coli*. **(A)** Microcin (Mcc) gene clusters in *Escherichia coli* CA58, H47 and Nissle 1917 (EcN) (modified from Vassiliadis G. et al., 2010). Mcc precursor genes, immunity genes, and genes involved in Mcc export and posttranslational modifications are indicated in yellow, red, blue and green, respectively. Truncated genes in *E*. *coli* H47 and EcN are shown with slashes. The names of the genes specifically involved in MccM, MccH47, and MccI47 are in red, pink, and blue respectively. **(B)** Phylogenetic relationships between *E*. *coli* strains that carry siderophore-Mcc, colibactin (*pks*) and salmochelin (*iro*) synthesis gene clusters. The phylogenetic group of the strains is shown together with the phylogeny that was determined based on the housekeeping gene *rpoC*. The presence of Mcc precursor genes (*mcmA*, *mchB*), immunity genes (*mcmI*, *mchI*), genes involved in Mcc export (*mchE*, *mchF*, *mcmM*) and genes involved in posttranslational modifications (*mcmL*, *mcmK*) are indicated with the same colour code as in Fig 7A.

We demonstrated that strains of *E*. *coli* that carry a truncated Mcc gene cluster exhibit a siderophore-Mcc-dependent antibacterial activity (Fig 7A). This antibacterial activity requires ClbP from the biosynthetic pathway that produces the genotoxin colibactin and IroB from the biosynthetic pathway that produces the siderophore salmochelin. Consequently, we checked this association between the *pks* island, the *iro* locus and the Mcc island in *E*. *coli* strains with genomes available in GenBank. Interestingly, all strains that lacked the *mcmL* and *mcmK* genes responsible for posttranslational modifications belonged to the B2 phylogroup and carried the *pks* island and *iroA* (Fig 7B), except for strain 1105 deprived of *pks* island. Conversely, the strains that carry *mcmL/mchA* and *mcmK/mchS1* belonged to B1, C or D phylogroups and lacked the *pks* island. These particular associations of genetic determinants led to the hypothesis that the truncated island is present almost exclusively in strains that carry *pks* and the *iroA* locus. It suggests that this interplay between colibactin, salmochelin, and the siderophore-Mcc biosynthetic pathways is due to a co-selection in strains that are either pathogenic or probiotic.

## Discussion

Since Fleming discovered penicillin in 1928, antibiotics have contributed to the increase in human life expectancy. Many infections which were previously fatal became curable. Unfortunately, the overuse and misuse of antibiotics, in parallel with the lack of new antibacterial drugs enabled multi-resistant bacteria to emerge and spread [50]. According to the World Health Organization (WHO), this phenomenon “poses a substantial threat to morbidity and mortality worldwide” [51]. The trend is especially worrying for Gram-negative bacteria. For instance, the number of deaths attributable to 3^rd^ generation cephalosporin-resistant or carbapenem-resistant *E*. *coli* increased by more than 4 times in Europe between 2007 and 2015 [52]. Of the antibiotics that are currently being developed for intravenous administration, only a small proportion (15 out of 44) demonstrates some activity against Gram-negative bacteria, and all these molecules are derived from known antibiotic classes. Consequently, the WHO established that research and development of new antibiotics against Gram-negative bacteria was a “critical priority” [51].

In the search for new antimicrobials, Mcc seem a promising alternative to “conventional” antibiotics. In fact, many Mcc exhibit potent narrow-spectrum antimicrobial activity, whereas antibiotics can eliminate beneficial bacteria, alter the microbiota and promote the selection of resistant strains [53,54]. A major challenge in using Mcc is their delivery in sufficient quantities to the site of infection, especially after oral administration because they are often degraded in the upper digestive tract [55,56]. Engineered probiotic bacteria were consequently proposed as *in situ* producers of Mcc to fight against enteropathogens [57] or to reduce colonization by multi-resistant bacteria [58].

EcN has been used as a probiotic for over a century, with numerous therapeutic benefits described. However, serious concerns about the safety of EcN administration have emerged over the years. EcN was reported to be responsible for severe sepsis in an infant [59] and its genome was shown to host the pathogenicity island *pks* [21,37], which codes for colibactin, a *bona fide* virulence factor for *E*. *coli* strains responsible for extraintestinal infections [26,27]. In addition, the carriage of colibactin producing *E*. *coli* could also be deleterious to gut homeostasis. In adult rats, it increased intestinal epithelial permeability and led to signs of genotoxic damages in intestinal cells, such as crypt fission and increased cell proliferation [28]. In mice predisposed to colorectal cancer, *pks-*positive *E*. *coli* increased the size and the number of tumors [33,60]. In human beings, several studies reported that *pks-*positive *E*. *coli* were over-represented in colorectal cancer biopsies compared to controls [33,34,61]. On a whole, these studies suggest that colibactin-producing bacteria could promote tumorigenesis. Therefore, our goal was to understand the interplay between the production of the genotoxin colibactin and the beneficial effects related to the *pks* island in the probiotic activity of EcN. Consequently, we attempted to disarm EcN while keeping its antagonistic property.

**Fig 8:**
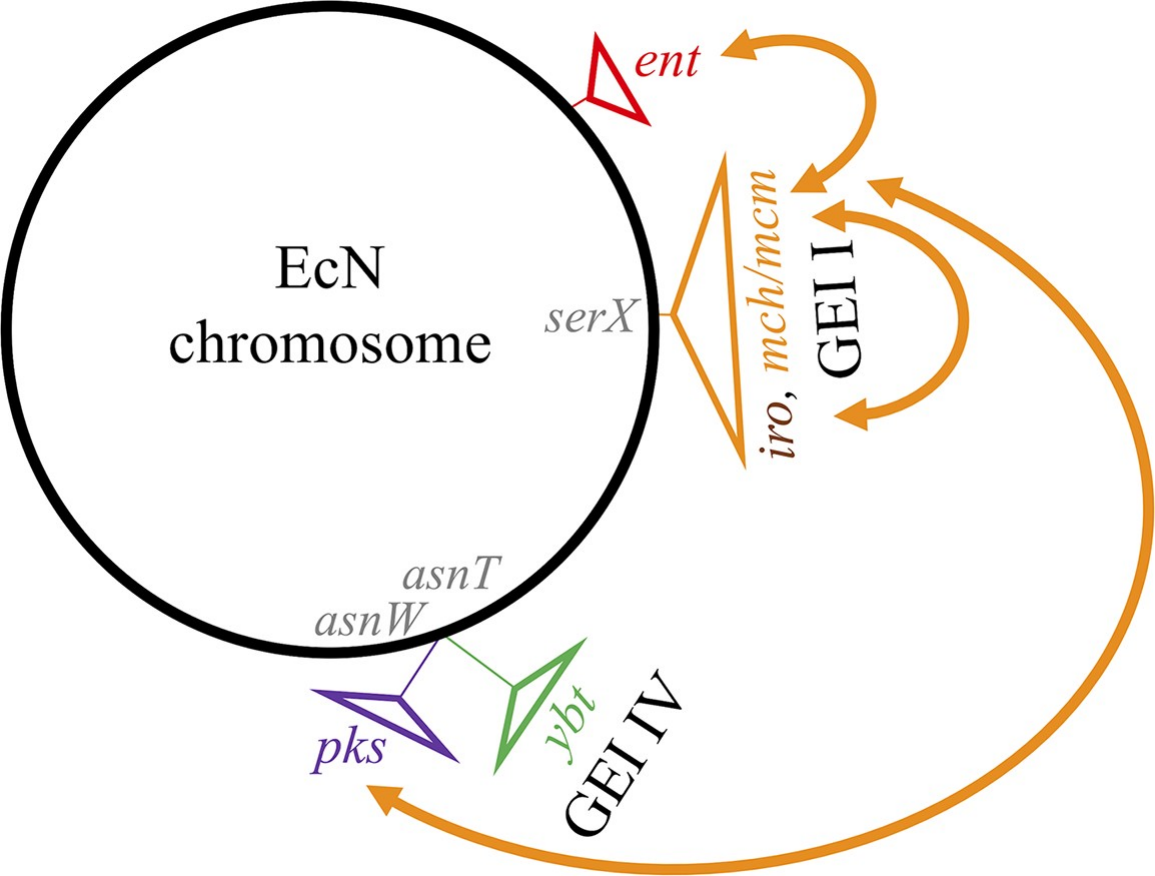
Gene clusters involved in the production of microcins H47 and M (MccH47 and M) in *E*. *coli* strain Nissle (EcN) represented on a genomic map. The loci that encode enterobactin (*ent*), colibactin (*pks*), yersiniabactin (*ybt*) on EcN genomic island (GEI) IV, salmochelin (*iro*) and MccH47 (*mch*) and M (*mcm*) on GEI I are represented. The arrows represent the interplays between the different gene clusters involved in MccH47 and M production in EcN.

In a previous attempt, our team constructed a non-genotoxic EcN PPTase ClbA mutant, which also lost its probiotic activity [37]. Subsequently, it was discovered that the PPTase ClbA contributes to the synthesis of enterobactin (and therefore salmochelin) and yersiniabactin [38]. In this study, we demonstrated that there is collaboration between the salmochelin (*iroB*) and the Mcc gene clusters, both of which are located on EcN genomic island I, and the *pks* island (*clbP*) (Fig 8). The interweaving is so strong between these determinants, that a single protein, ClbP is involved both in colibactin and Mcc production. Up until now, ClbP had only been described as a peptidase that removes the C14-asparagine prodrug scaffold from precolibactin [24,25]. Although the complete C-terminal domain with the three transmembrane helices is required for the bioactivity of ClbP, the catalytic activity is performed by the N-terminal periplasmic domain [24,46]. In this study, we demonstrated that the C-terminal domain of ClbP, deprived of known enzymatic function, is necessary for EcN antagonistic activity due to MccH47 and MccM.

**Fig 9:**
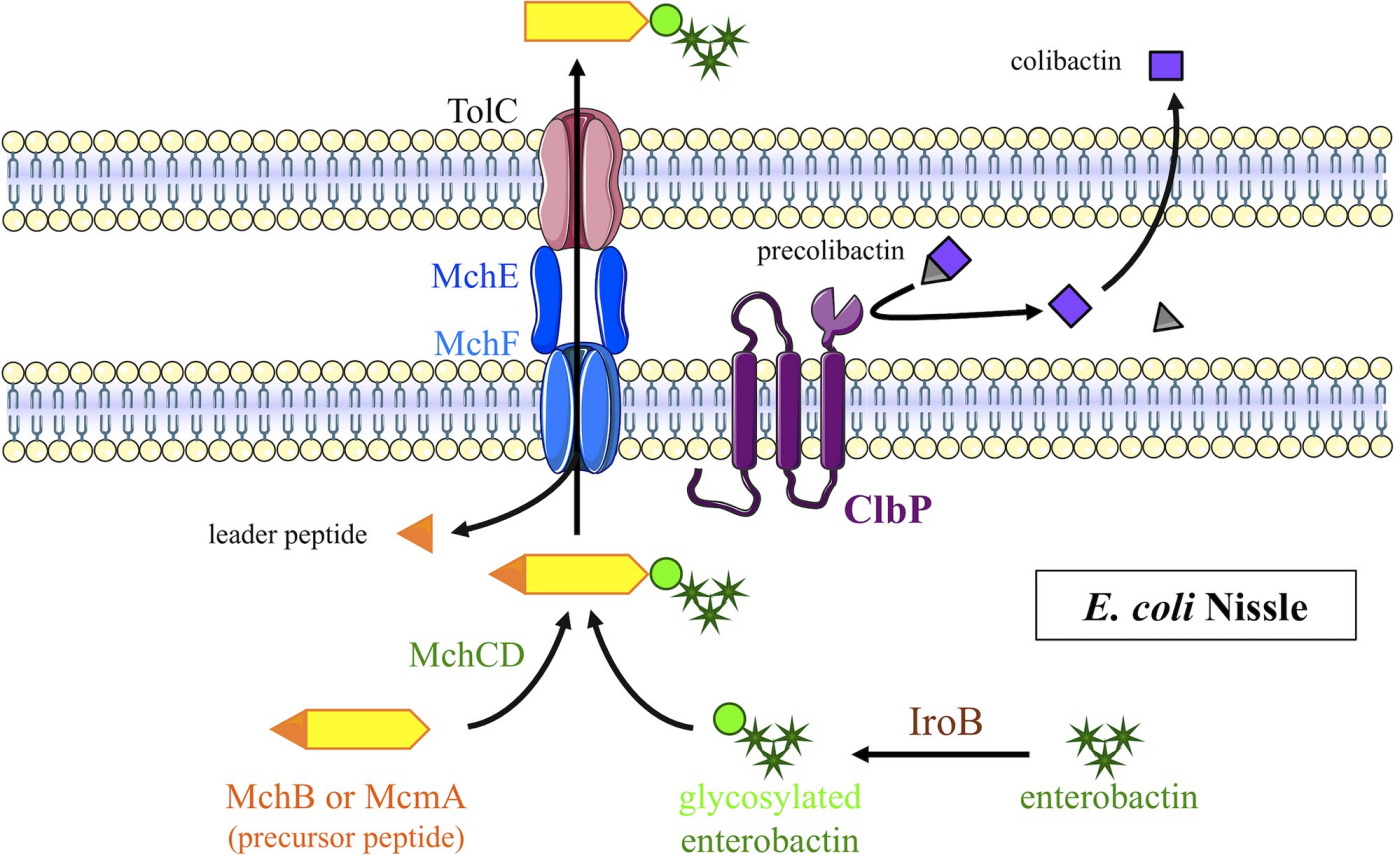
Model proposed for biosynthesis of siderophore-microcins H47 and M in *E*. *coli* Nissle. An enterobactin precursor is modified by the enterobactin glucosyltransferase IroB. This siderophore moiety is transferred onto the C-terminal extremity of the precursor peptide by the MchCD complex. The active form of the siderophore-microcin is the result of the leader peptide cleavage during its export by the specific efflux pump MchEF-TolC. The C-terminal transmembrane domain of ClbP allows this final step of siderophore-microcin production, while its N-terminal enzymatic domain cleaves precolibactin to produce colibactin.

Whereas IroB seems to replace the enterobactin glucosyltransferase McmL absent in EcN, the McmK homolog IroD is not necessary for EcN antagonistic activity. In the absence of another enterobactin esterase homolog, we can speculate that the enterobactin moiety of EcN Mcc is not linearized but remains cyclic. Besides, the protein complex MceIJ, which is the homolog of EcN MchCD in *K*. *pneumoniae* strain E492, recognizes and links both the cyclic and linearized glucosylated derivatives of enterobactin [45]. Romano et al. recently demonstrated that the ATP-binding cassette exporter McjD is highly specific of the Mcc J25 [62]. The ATP-binding cassette exporter MchEF is conserved among MccH47 and MccM producing strains whether the microcin gene cluster is “complete” or not (*e*.*g*. with differences of only 1.2% (n = 5) for MchE and 1.3% (n = 9) for MchF at the amino-acid level between EcN and *E*. *coli* H47 which carries a “complete” siderophore-Mcc gene cluster). In EcN, we showed that overexpression of this pump abolished the ClbP-dependence of EcN antagonistic effect, and that hetero-complementation with “ClbP-like” proteins restored the EcN antibacterial effect. We thus propose that the ClbP C-terminal transmembrane domain could facilitate the export of the siderophore-Mcc (Fig 9).

A recent study demonstrated that the Mcc are a key factor for EcN to outcompete *S*. Typhimurium following co-administration in mice [4]. In our study, using a similar mice infection model, we observed that deletion of *clbP* resulted in the partial loss of protection against *S*. Typhimurium. In contrast, the single nucleotide mutation *clbP*-S95R was as efficient as the wild-type probiotic to outcompete *S*. Typhimurium and to decrease the clinical symptoms of salmonellosis. Taking together with our *in vitro* data, this suggests that EcN needs both the Mcc and ClbP, but not mature colibactin or C14-asparagine, for protection against *S*. Typhimurium *in vivo*. The EcN *clbP-*S95R did not induce any sign of genotoxicity in infected cultured cells. This opens a way for the engineering of an EcN-derived probiotic that could be used safely.

Using both functional and bioinformatic analyses, we demonstrated the interplay between siderophore-Mcc, salmochelin, and colibactin synthesis pathways. Strikingly, two groups of *E*. *coli* strains emerged. On one hand, all strains that carry a “truncated” MccH47 and MccM gene cluster (*i*.*e*. strains such as EcN lacking *mcmL/mchA* and *mcmK/mchS1*) are B2 strains that also bear the *pks* island and the *iroA* locus. It should be noted that isolates from urine were over-represented in this group of strains (CFT073, clones D i14 and D i2, UPEC 26–1, and ABU 83972). On the other hand, the *pks* island and the *iroA* locus are absent in the non-B2 strains that carry a “complete” MccH47 and MccM gene cluster. All these strains were isolated from stools (except ACN002 for which the origin is unknown). Therefore, we can hypothesize that these strains with a “complete” Mcc gene cluster are specialized in Mcc production in order to survive in the competitive intestinal environment, which is their exclusive niche. In contrast, extraintestinal pathogenic *E*. *coli* (ExPEC) must be efficient gut colonizers in order to emerge from the intestinal niche and infect other body sites (such as the urinary tract) to which they must subsequently adapt. That is why it has been suggested that ExPEC are “generalists” rather than specialized strains [63]. The strains we examined in our study fit this model. They can express various virulence factors depending on their environment: MccH47 and M, siderophores and analgesic lipopeptides derived from the colibactin pathway, for instance. To be able to produce so many virulence or fitness factors with a genome of limited size [64], the elements of the assembly lines that produce these determinants must be versatile and intervene in several apparently independent metabolic pathways.

In conclusion, we discovered that the *pks* island is even more intimately connected to EcN probiotic activity than expected. This entanglement probably reflects the co-evolution of probiotic, fitness and pathogenic determinants to adapt to various environments. Decoupling the antagonistic from the genotoxic activities by specifically targeting the enzymatic domain of ClbP opens the way to safe use of EcN.

## Materials and methods

### Bacterial strains, mutants and plasmids

The bacterial strains and plasmids used in this study are listed in S1 Table. Gene mutagenesis was performed by the lambda Red recombinase method [65] with the primers listed in S2 Table. The double mutants were constructed sequentially. The mutations and deletion of FRT cassettes were verified by PCR using primers upstream and downstream of the target genes.

The fusion between ClbP N-terminal signal sequence, the alkaline phosphatase PhoA, and the three transmembrane helices of ClbP were constructed using the HiFi DNA assembly kit (New England Biolabs, Ipswich, MA, USA) with primers overlapping between each fragment (S2 Table). The constructions were verified by PCR and confirmed by sequencing. The blue-stained colony-forming units on LB plates with 40 mg/L of 5-bromo-4-chloro-3-indolyl phosphate revealed the presence of the PhoA alkaline phosphatase domain in the periplasm as previously reported [46].

To construct plasmids p*mchEF* and p*mchCD*, the genes were PCR-amplified using primers listed in S2 Table and cloned into pCR-XL-TOPO (Invitrogen, Carlsbad, CA, USA).

To construct plasmid pIroB, the *iroB* gene was PCR-amplified with EcN genomic DNA as a template and primers IRSDNG7 and IRSDNG8, digested by EcoRI and BamHI and ligated into pBbA5a-RFP (obtained from Addgene) digested to remove the *rfp* gene.

The strain EcN *clbP-*S95R chromosomal isogenic mutant was constructed using a genome editing technique [66]. EcN was transformed with pORTMAGE, then grown in LB at 30°C and 300 rpm to reach OD600 = 0.5. An initial mutagenesis cycle was started by inducing the expression of Lambda recombinases and the dominant negative *mutL*E32K allele at 42°C for 15 minutes at 250 rpm. The culture was then cooled to 0°C, washed in water and electroporated with 50 μM of oligonucleotide IRSDNG26 that includes the S95R mutation in the *clbP* gene sequence. In a control experiment, the *lacZ* gene was targeted by a specific mutagenic oligonucleotide. Following recovery in LB at 30°C and 300 rpm for 1 hour, two other mutagenesis cycles were performed, and the bacteria were finally plated on MacConkey agar without any antibiotic. Approximately 33% of the isolates were LacZ negative in the control experiment. Sixty candidate *clbP-*S95R mutants were tested for loss of genotoxicity and megalocytosis phenotype in infected HeLa cells as previously described [67]. Non genotoxic mutants that had lost the pORTMAGE plasmid were selected, and were finally verified for removal of a ClaI restriction site by S95R mutation in the PCR amplified *clbP* sequence.

### Determination of the genotoxic effect induced by colibactin

The cellular senescence induced by colibactin was with the associated cell enlargement called megalocytosis and was determined for every EcN mutant constructed in this study in the Mcc gene cluster, in the *iroA* locus, and for the *clbP-*S95R mutant. As previously described [67], HeLa cells (ATCC, CCL-2) were infected for 4 hours. The cells were then washed and incubated with gentamicin for 72 hours before staining with Giemsa. The genotoxicity of EcN and the *clbP-*S95R chromosomal mutant was confirmed by an In-Cell Western procedure, as previously described [38]. In brief, HeLa cells were infected in 96-well plates for 4 hours at a given multiplicity of infection (number of bacteria per cell at the onset of infection). Four hours after the end of infection cells were fixed, permeabilized and stained with rabbit monoclonal anti-gamma-H2AX (Cell Signaling, 20E4, 1:200) followed by an infrared fluorescent secondary antibody. DNA was counterstained with RedDot2 (Biotum). Fluorescence was recorded with an Odyssey infrared imaging system (Li-Cor).

### Quantification of C14-asparagine

Bacterial pellets were suspended in 300μL HBSS and frozen at -80°C until lipids extraction. These samples were added with 5 μL of internal standard mixture (Deuterium-labeled compounds, 400 ng/mL) and crushed with 2x30 sec shaking at 5 m/s (Matrix A tubes, FastPrep, MP Biomedical). 300μL of cold methanol was added, the tubes were centrifuged at 1016 × g for 15 min (4°C) and the supernatants were stored at -80°C until extraction. Lipids were extracted on solid phase HLB 96 wells plates (OASIS HLB, Waters) conditioned with 500μL methanol and 500μL 90:10 water/methanol. Samples were brought to 2mL with water and loaded at one drop per 2 s, then washed with 500μL 90:10 water/methanol and dried under aspiration. Lipids were eluted with 750μL methanol and evaporated twice under N_2_, then suspended in 10 μL methanol. The quantification of C14-asparagine was performed by the MetaToul Lipidomics facility (Inserm UMR1048, Toulouse, France), using an in-house quantification assay by high-performance liquid chromatography/tandem mass spectrometry analysis [36].

### Competitive growth assay

Strains were grown in lysogeny broth (LB Lennox, Invitrogen) overnight at 37°C with shaking at 240 rpm. Rifampicin, streptomycin, kanamycin, carbenicillin or chloramphenicol was added as required to the medium.

The media used for co-culture experiments were either M63 minimal medium with final concentrations of 15 mM ammonium sulfate, 1 mM magnesium sulfate heptahydrate, 100 mM monopotassic phosphate, 2.5 g/L glucose, 1 mg/L thiamine, and 1 g/L Bacto tryptone (BD Biosciences, Le Pont de Claix, France), or Dulbecco’s Modified Eagle Medium (DMEM) GlutaMAX (Invitrogen) supplemented with 25 mM Hepes, 10% (v/v) Fetal Calf Serum (FCS, Eurobio, Courtaboeuf, France), and 1% (v/v) Non Essential Amino Acids (NEAA, Invitrogen).

500 μL of each overnight culture were cultured in 9.5 mL of co-culture medium and incubated for 2h at 37°C with shaking at 240 rpm. Both the producing and the target strains (EcN and LF82 respectively) were inoculated from these 2-hour-cultures at 10^6^ CFU/mL in 10 mL of co-culture medium as previously described [3] and incubated for 24h at 37°C with shaking at 240 rpm. For CFU numeration, the culture broth was serial-diluted in PBS and plated on selective LB agar plates containing the antibiotic required (*e*.*g*. rifampicin for LF82 [68]). In the total results section, only the growth of the target strains (mainly LF82) is reported. As a control, the growth of the competitive strains (mainly EcN and EcN mutants) was systematically checked (S9 Fig).

### Animal infections

The animal infections were performed following the European directives for the protection of animal used for scientific purposes (2010/63/EU). The protocol was approved by a local ethic committee (number of protocol: 2019070811071023). Female C57BL/6 (Janvier) were housed in ventilated cages, 5 animals per cage, with *ad libitum* access to food and water. The animals were administered by oral gavage 20 mg of streptomycin, then 24h later, infected *per os* with 10^9^ *S*. Typhimurium strain IR715 (nalidixic acid resistant) or co-administered with 10^9^ *S*. Typhimurium and 10^9^ EcN, EcN Δ*clbP* or EcN *clbP*S95R (with the *rpsL*K42R allele to confer resistance to streptomycin). Fecal shedding of *S*. Typhimurium and EcN was quantified by plating of homogenized feces on LB agar supplemented with nalidixic acid or streptomycin. The severity of the salmonellosis was evaluated by daily scoring of weight loss, signs of abdominal pain, fever and diarrhea. The experiment was terminated at 4 days after infection to avoid lethality. The experiment was repeated three times with five animals per group, and the clinical score was scored blindly (without knowledge of the infecting bacteria) in two out of the three independent experiments.

### Bioinformatic analysis

Genes involved in MccH47 and MccM synthesis were searched using BLASTn and the CA58 Mcc gene cluster as a reference: *mchB* and *mcmA* which encode precursor proteins, the immunity genes *mchI* and *mcmI*, genes *mchE* and *mchF* which encode a specific efflux pump, and genes *mcmK* and *mcmL* (and their respective homologs in the *E*. *coli* H47 Mcc gene cluster, *mchS1* and *mchA*) responsible for posttranslational modifications. A query cover > 80%, an identity > 90%, and an E-value < 1e-40 were chosen as cutoff values for significance. The genes *clbB* and *clbP*, as respective markers for the 5’ and 3’ regions of the *pks* island, were identified using the same method, and so were genes *iroN* and *iroB* as markers for the 5’ and 3’ regions of the salmochelin gene cluster (*iroA* locus). Phylogroups were determined *in silico* based on the presence/absence of 4 genes: *arpA*, *chuA*, *yjaA*, *and tspE4*.*C2* (and *trpA* to distinguish the A and C phylogroups) [69]. The phylogenetic tree was constructed with the *rpoC* sequence. The sequences were collected using PATRIC 3.5.8 [70], aligned by multiple sequence comparison by log-expectation (MUSCLE) with the MEGA7.0.26 software [71], and the phylogenetic tree was constructed according to the maximum likelihood method with MEGA7.0.26.

### Statistical analyses

Statistical analyses were carried out using GraphPad Prism 7.0a (GraphPad, San Diego, CA, USA). *P* values were calculated using one-way ANOVA followed by Bonferroni post-tests. CFU/ml were log-transformed for the analyses. *P* values < 0.05 were considered significant and are denoted by ★, *P*<0.01 is denoted by ★★, and *P*<0.001 by ★★★.

## Supporting information

S1 Table Strains and plasmids used in this study. (DOCX)

S2 Table Primers used in this study. (DOCX)

S3 Table Genbank accession numbers for genome sequences included in Fig 7B. (DOCX)

S1 Fig *In vitro* growth curves of *E*. *coli* LF82 when grown alone or in competition with *E*. *coli* Nissle wild type or Δ*clbP* mutant. Colony forming unit (CFU) counts of *E*. *coli* LF82 following a 2, 4, 6, 8, and 24-hour co-culture in M63 medium with the wild-type (WT) *E*. *coli* strain Nissle 1917 (EcN) or the mutant for colibactin maturing peptidase ClbP (Δ*clbP*). LF82 was also cultured alone as a control. The individual results of the 3 independent experiments are shown and the medians for each time point are linked.
(TIF)

S2 Fig *E*. *coli* Nissle ClbP-dependent antagonistic activity against phylogenetically-related enterobacteria. Colony forming unit (CFU) counts of *E*. *coli* ST131 isolate JJ1886, *E*. *coli* NRG857c, *Salmonella enterica* serovar Typhimurium IR715, *Enterobacter aerogenes* ATCC^®^13048, and *Klebsiella oxytoca* ATCC^®^13182 after a 24-hour co-culture in Dulbecco’s Modified Eagle Medium with wild-type (WT) *E*. *coli* strain Nissle (EcN), and EcN Δ*clbP* mutant. All susceptible strains were also cultured alone as controls (∅). The medians and individual results of independent experiments are shown. One-way ANOVA and Bonferroni post-tests; ★★★P<0.001.
(TIF)

S3 Fig Cytopathic activity of *E*. *coli* Nissle mutants in the microcin and salmochelin production systems, and in ClbP catalytic site. HeLa cells were transiently infected with wild-type (WT) *E*. *coli* strain Nissle (EcN) (B) and various mutants then washed and incubated with gentamicin for 72 hours before staining with Giemsa. The control is shownd in A. Bars represent 50 μm. Mutations in the EcN microcin gene cluster did not abrogated EcN cytopathic activity: EcN mutants for C. *mcmA;* D. *mchB*; E. *mcmAmchB*; F. *mchCD*; G. *mchEF*. Mutations in the EcN salmochelin gene cluster did not abrogated EcN cytopathic activity: EcN mutants for H. *iroB*; I. *iroC*; J. *iroD*; K. *iroE*. EcN Δ*clbP* mutant (L) did not affect cells, whereas *clbP* complementation restored the cytopathic activity (M). Complementation of EcN Δ*clbP* mutant with a plasmid encoding ClbP with an S95A mutation (N), a K98T mutation (O) did not restore the cytopathic activity, as well as complementation with the plasmid encoding the fusion between alkaline phosphatase and the C-terminal domain of ClbP (P).
(TIF)

S4 Fig *E*. *coli* Nissle antagonistic activity against LF82 carrying microcin M (Fig S4A) or microcin H47 (Fig S4B) immunity genes. Colony forming unit (CFU) counts of *E*. *coli* LF82 carrying; A: a plasmid encoding microcin M (MccM) immunity gene *mcmI* (pMcMi); B: microcin H47 (MccH47) immunity gene *mchI* (pMcHi) after a 24-hour co-culture in M63 minimal medium with wild-type (WT) *E*. *coli* strain Nissle (EcN), EcN mutants for MccM precursor gene *mcmA*, for MccH47 precursor gene *mchB*, and for both *mcmA mchB* genes. LF82 carrying pMcMi or pMcHi were also cultured alone as controls (∅). The medians and individual results of independent experiments are shown. One-way ANOVA and Bonferroni post-tests in comparison with co-culture with WT; ★★★P<0.001.
(TIF)

S5 Fig Antagonistic activity against LF82 of *E*. *coli* Nissle Δ*clbP* mutant complemented with ClbP orthologs. Colony forming unit (CFU) counts of *E*. *coli* LF82 following a 24-hour-coculture in M63 medium with wild-type (WT) *E*. *coli* strain Nissle 1917 (EcN), the *clbP* mutant and complemented strain with a plasmid encoding wild-type ClbP (p*clbP*), with plasmids encoding ClbP-like proteins from *Bacillus weihenstephanensis* (Bwei), *Hahella chejuensis* (Hche), *B*. *mycoides* (Bmyc), *B*. *pseudomycoides* (Bpse), and *Clostridium cellulolyticum* (Ccel). LF82 was also cultured alone as a control (∅). Medians and individual results of independent experiments are shown. One-way ANOVA and Bonferroni post-tests in comparison with coculture with WT; ★★★P<0.001.
(TIF)

S6 Fig Impact of *mchEF* or *clbP* overexpression on *E*. *coli* Nissle antagonistic activity against LF82. Colony forming unit (CFU) counts of *E*. *coli* LF82 following a 24-hour co-culture in M63 medium with WT EcN, EcN mutant for the peptidase ClbP and the corresponding mutant overexpressing the efflux pump MchEF (p*mchEF*), EcN mutant for MchEF and the corresponding mutant overexpressing ClbP (p*clbP*). LF82 was also cultured alone as a control (∅). The medians and individual results of independent experiments are shown. One-way ANOVA and Bonferroni post-tests in comparison with co-culture with WT; ★★★P<0.001.
(TIF)

S7 Fig C14-asparagine produced by *E*. *coli* Nissle and *clbP-*S95R mutant. *E*. *coli* Nissle (EcN), a *clbN* deletion mutant and the genome edited *clbP*-S95R mutant were grown 24 h in DMEM in triplicate cultures, then the bacteria were pelleted, the lipids were extracted and the colibactin cleavage product C14-Asn was quantified by liquid chromatography coupled to mass spectrometry. The results of the triplicate tubes and median are shown. Nd: none detected.
(TIF)

S8 Fig ClbP-dependent antagonist activity of strains carrying both a truncated microcin gene cluster and the *pks* island. Colony forming unit (CFU) counts of *E*. *coli* LF82 following a 24-hour co-culture in Dulbecco’s Modified Eagle Medium with *E*. *coli* strains that carry both a truncated microcin gene cluster and the *pks* island: wild-type (WT) *E*. *coli* strains Nissle 1917 (EcN), CFT073 (CFT), ABU83972 (ABU), ATCC 25922 (ATCC), the corresponding *clbP* gene deletion mutants and complemented mutant strains, and *pks+ E*. *coli* strain M1/5 (human commensal), SP15 (responsible for meningitis), NC101 (murine commensal and procarcinogenic), MG1655 that hosts a bacterial artificial chromosome (BAC) bearing the *pks* island (MGbacpks), and the corresponding *clbP* gene deletion mutants. LF82 was also cultured alone as a control (∅). Medians and individual results of independent experiments are shown. One-way ANOVA and Bonferroni post-tests; ★★★P<0.001.
(TIF)

S9 Fig Growth of EcN and its mutants in co-culture experiments. Colony forming unit (CFU) counts of EcN and its mutants following a 24-hour co-culture in Dulbecco’s Modified Eagle Medium with *E*. *coli* LF82.
(TIF)
