# Supplementary material for: Deciphering the interplay between the genotoxic and probiotic activities of Escherichia coli Nissle 1917
Source: PLoS Pathog. 2019 Sep 23;15(9):e1008029. doi: 10.1371/journal.ppat.1008029 (PMC6776366; doi:10.1371/journal.ppat.1008029)
Supplement: S1 Table — (DOCX) [file ppat.1008029.s001.docx]

**Table S1.** **Strains and plasmids used in this study**.

| **Strain or plasmid** | **Genotype or phenotype** | **Source or reference** |
| --- | --- | --- |
| *E. coli* Nissle (EcN) | Probiotic strain; colibactin genotoxin producer; enterobactin and salmochellin siderophores producer; microcins H47 and M producer | DSM 6601, Mutaflor^®^ |
| EcN WT | EcN mutant in *rpsl*, Str^R^ | (1,2) |
| EcN Δ*clbA* | *clbA* mutant of strain EcN WT, Str^R^, Kan^R^ | (1) |
| EcN Δ*clbB* | *clbB* mutant of strain EcN WT, Str^R^, Kan^R^ | (3) |
| EcN Δ*clbC* | *clbC* mutant of strain EcN, Str^R^, Chl^R^ | (3) |
| EcN Δ*clbH* | *clbH* mutant of strain EcN WT, Str^R^, Kan^R^ | This study |
| EcN Δ*clbL* | *clbL* mutant of strain EcN WT, Str^R^, Kan^R^ | This study |
| EcN Δ*clbM* | *clbM* mutant of strain EcN WT, Str^R^, Kan^R^ | This study |
| EcN Δ*clbN* | *clbN* mutant of strain EcN WT, Str^R^, Kan^R^ | (3) |
| EcN Δ*clbO* | *clbO* mutant of strain EcN WT, Str^R^, Kan^R^ | This study |
| EcN Δ*clbP* | *clbP* mutant of strain EcN WT, Str^R^, Kan^R^ | (3) |
| EcN Δ*clbP* p*clbP* | EcN Δ*clbP* mutant complemented with pBRSKp*clbP*, Str^R^, Kan^R^, Carb^R^ | This study |
| EcN Δ*clbP* pclbP-S95A | EcN Δ*clbP* mutant complemented with pclbP-S95A, Str^R^, Kan^R^, Carb^R^ | This study |
| EcN Δ*clbP* pclbP-K98T | EcN Δ*clbP* mutant complemented with pclbP-K98T, Str^R^, Kan^R^, Carb^R^ | This study |
| EcN Δ*clbP* pclbP-3H | EcN Δ*clbP* mutant complemented with pclbP-3H, Str^R^, Kan^R^, Carb^R^ | This study |
| EcN Δ*clbP* Bwei | EcN Δ*clbP* mutant complemented with p33Bweihenstephanensis, Str^R^, Kan^R^, Carb^R^ | This study |
| EcN Δ*clbP* Hche | EcN Δ*clbP* mutant complemented with p33Hchejuensis, Str^R^, Kan^R^, Carb^R^ | This study |
| EcN Δ*clbP* Ccel | EcN Δ*clbP* mutant complemented with p33Ccellulolyticum, Str^R^, Kan^R^, Carb^R^ | This study |
| EcN Δ*clbP* Bmyc | EcN Δ*clbP* mutant complemented with p33Bmycoides, Str^R^, Kan^R^, Carb^R^ | This study |
| EcN Δ*clbP* Bpse | EcN Δ*clbP* mutant complemented with p33Bpseudomycoides, Str^R^, Kan^R^, Carb^R^ | This study |
| EcN *clbP-*S95R | EcN *clbP-*S95R chromosomal isogenic mutant, Str^R^ | This study |
| EcN Δ*clbP*::FRT | *clbP*::FRT mutant of strain EcN WT, Str^R^ | This study |
| EcN Δ*clbP* p*mchEF* | EcN Δ*clbP*::FRT carrying TopoXL *mchEF,* Str^R^, Kan^R^ | This study |
| EcN Δ*clbQ* | *clbQ* mutant of strain EcN WT, Str^R^, Kan^R^ | This study |
| EcN Δ*mcmA* | *mcmA* mutant of EcN WT, Str^R^, Kan^R^ | This study |
| EcN Δ*mchB* | *mchB* mutant of EcN WT, Str^R^, Kan^R^ | This study |
| EcN Δ*mcmA*Δ*mchB* | *mcmA mchB* mutant of EcN WT, Str^R^, Kan^R^ | This study |
| EcN Δ*mchCD* | *mchC mchD* mutant of EcN WT, Str^R^, Kan^R^ | This study |
| EcN Δ*mchCD*::FRT | *mchCD*::FRT mutant of strain EcN WT, Str^R^ | This study |
| EcN Δ*mchCD* p*mchCD* | EcN Δ*mchCD*::FRT complemented with TopoXL *mchCD,* Str^R^, Kan^R^ | This study |
| EcN Δ*mchEF* | *mchE mchF* mutant of EcN WT, Str^R^, Kan^R^ | This study |
| EcN Δ*mchEF* p*clbP* | EcN Δ*mchEF* carrying pBRSKp*clbP*, Str^R^, Kan^R^, Carb^R^ | This study |
| EcN Δ*mchEF*::FRT | *mchEF*::FRT mutant of strain EcN WT, Str^R^ | This study |
| EcN Δ*mchEF* p*mchEF* | EcN Δ*mchEF*::FRT complemented with TopoXL *mchEF,* Str^R^, Kan^R^ | This study |
| EcN Δ*entE* | *entE* mutant of EcN WT, Str^R^, Chl^R^ | This study |
| EcN Δ*entD* | *entE* mutant of EcN WT, Str^R^, Chl^R^ | This study |
| EcN Δ*entD*Δ*clbA* | *clbA, entE* mutant of EcN WT, Str^R^, Kan^R^ | This study |
| EcN Δ*iroB* | *iroB* mutant of EcN WT, Str^R^, Chl^R^ | This study |
| EcN Δ*iroB* p*iroB* | EcN Δ*iroB* mutant complemented with pASK75 iroB | This study |
| EcN Δ*iroC* | *iroC* mutant of EcN WT, Str^R^, Kan^R^ | This study |
| EcN Δ*iroD* | *iroD* mutant of EcN WT, Str^R^, Chl^R^ | This study |
| EcN Δ*iroE* | *iroE* mutant of EcN WT, Str^R^, Kan^R^ | This study |
| *E. coli* LF82 | Strain isolated from an ileal biopsy of a patient with Crohn’s disease; adherent-invasive *E. coli,* Rif^R^ | (4) |
| LF82 pMcMi | LF82 carrying pMcMi, Rif^R^, Carb^R^ | This study |
| LF82 pMcHi | LF82 carrying pMcHi, Rif^R^, Carb^R^ | This study |
| *E. coli* M1/5 | Commensal *E. coli* strain isolated from feces of a healthy adult;  B2 phylogenetic group; colibactin genotoxin producer;  aerobactin, enterobactin and yersiniabactin siderophores producer | (5) |
| M1/5 Δ*clbP* | *clbP* mutant of strain M1/5, Kan^R^ | (6) |
| *E. coli* SP15 | Strain isolated from spinal fluid of a neonate with meningitis;  O18:K1 serotype; colibactin genotoxin producer;  aerobactin, enterobactin, salmochelins and yersiniabactin siderophores producer | (7) |
| SP15 Δ*clbP* | *clbP* mutant of strain SP15, Kan^R^ | This study |
| *E. coli* NC101 | Non-pathogenic murine *E. coli* strain; colibactin genotoxin producer; | (8,9) |
| NC101 Δ*clbP* | *clbP* mutant of strain NC101, Kan^R^ | (10) |
| *E. coli* MG1655 bac*pks* | Enterobactin siderophore producer *E. coli* strain carrying a bacterial artificial chromosome bearing the entire *pk*s island, Chl^R^ | (11) |
| MG1655 bac*pks* Δ*clbP* | *clbP* mutant of strain MG1655 bac*pks*, Chl^R^ | This study |
| *E. coli* CFT073 | Strain isolated from a patient with pyelonephritis, colibactin genotoxin producer;  enterobactin siderophores producer; microcins H47 and M producer | (12) |
| CFT073 Δ*clbP* | *clbP* mutant of strain CFT073, Kan^R^ | This study |
| CFT Δ*clbP* p*clbP* | CFT073 Δ*clbP* mutant complemented with pBRSKp*clbP*, Kan^R^, Carb^R^ | This study |
| *E. coli* ABU83972 | Strain isolated from a patient with asymptomatic bacteriuria, colibactin genotoxin producer;  enterobactin siderophores producer; microcins H47 and M producer | (13,14) |
| ABU83972 Δ*clbP* | *clbP* mutant of strain ABU83972, Kan^R^ | This study |
| ABU83972 Δ*clbP* p*clbP* | ABU83972 Δ*clbP* mutant complemented with pBRSKp*clbP*, Kan^R^, Carb^R^ | This study |
| *E. coli* ATCC^®^25922 | Strain isolated from a patient in Seattle (1946), colibactin genotoxin producer;  enterobactin siderophores producer; microcins H47 and M producer | DSM1103 |
| ATCC^®^25922 Δ*clbP* | *clbP* mutant of strain ATCC^®^25922, Kan^R^ | This study |
| ATCC^®^25922 Δ*clbP* p*clbP* | ATCC^®^25922 Δ*clbP* mutant complemented with pBRSKp*clbP*, Kan^R^, Carb^R^ | This study |
| *E. coli* ST131 isolate JJ1886 | Strain isolated in the USA (2007) from a patient with fatal urosepsis, Str^R^, Kan^R^, Carb^R^, Chl^R^ | (15) |
| *E. coli* NRG857c | Strain isolated from the ileum of a Crohn's Disease patient, Carb^R^, Chl^R^ | (16) |
| *Salmonella enterica* serovar Typhimurium IR715 | Nal^R^ derivative of *S. enterica* serovar Typhimurium ATCC14028 | (17) |
| *Enterobacter aerogenes* ATCC^®^13048 | Strain isolated from sputum in the USA (Center for Disease Control and Prevention) | ATCC^®^13048 |
| *Klebsiella oxytoca* ATCC^®^13182 | Strain isolated from a pharyngeal tonsil | ATCC^®^13182 |
| p*clbP* | pBRSK encoding *clbP* sequence | (18) |
| p*clbP*-S95A | pBRSK encoding the mutant S95A of ClbP (pOB902), Carb^R^ | (18) |
| p*clbP*-K98T | pBRSK encoding the mutant K98T of ClbP (pOB903), Carb^R^ | (18) |
| p*clbP*-3H | pASK74 carrying the fusion between ClbP N-terminal signal sequence, the alkaline phosphatase PhoA, and the 3 transmembrane helices of ClbP, Carb^R^ | This study |
| p33Bweihenstephanensis | pASK-IBA33plus encoding ClbP-like-encoding sequence of the locus tag *BcerKBAB4_1783* without the putative ‘half-size’ ABC-type exporter domain, Carb^R^ | (18) |
| p33Hchejuensis | pASK-IBA33plus encoding ClbP-like-encoding sequence of the locus tag *HCH_03446* without the putative ‘half-size’ ABC-type exporter domain, Carb^R^ | (18) |
| p33Ccellulolyticum | pASK-IBA33plus encoding ClbP-like-encoding sequence of the locus tag *Ccel_2389* without the putative ‘half-size’ ABC-type exporter domain, Carb^R^ | (18) |
| p33Bmycoides | pASK-IBA33plus encoding ClbP-like-encoding sequence of the locus tag *bmyco0001_51990,* Carb^R^ | (18) |
| p33Bpseudomycoides | pASK-IBA33plus encoding ClbP-like-encoding sequence of the locus tag *bpmyx0001_2150,* Carb^R^ | (18) |
| p*mchCD* | pCR XL-TOPO vector encoding *mchC* and *mchD* from EcN, Kan^R^ | This study |
| p*mchEF* | pCR XL-TOPO vector encoding *mchE* and *mchF* from EcN, Kan^R^ | This study |
| pMcMi | Carrying *mcmI* from MccM gene cluster, Carb^R^ | F. Moreno, unpublished data (19) |
| pMcHi | Carrying *mchI* from MccH47 gene cluster, ChlR | F. Moreno, unpublished data, (19) |
