# Supplementary material for: Deciphering the interplay between the genotoxic and probiotic activities of Escherichia coli Nissle 1917
Source: PLoS Pathog. 2019 Sep 23;15(9):e1008029. doi: 10.1371/journal.ppat.1008029 (PMC6776366; doi:10.1371/journal.ppat.1008029)
Supplement: S2 Table — (DOCX) [file ppat.1008029.s002.docx]

**Table S2.** **Primers used in this study.**

| **Primer** | **Aim** | **Primer sequence (5’-3’)** |
| --- | --- | --- |
| dmcmA_F | Delete *mcmA* | TTAAACGTGAATTTTTACTTCTTCACAAATCTTATAGCGAAGGTGTTGAAGCTGGAGCTGCTTC |
| dmcmA_R |  | TATCATATAATGAAGCACCTGTAACAGGTGCTTCATTAACAATATAAGGTCATATGAATATCCTCCTTAG |
| VmcmA_F | Verify *mcmA* deletion | CGTTGAGAACTGTTGACAGG |
| VmcmA_R |  | AAGCGTTACATAGGCACCAT |
| dmchB_F | Delete *mchB* | TCTGGTCTGGTCAGGCTGGAAAAACGGAAGTTAAATATGATGGAGTTTATGCTGGAGCTGCTTC |
| dmchB_R |  | ATTTTACAGGTACTACCGAAAAAGTACCTGTAAAATAAATAAAAATATAACATATGAATATCCTCCTTAG |
| VmchB_F | Verify *mchB* deletion | GTTTCACTTTCATCCCTTCG |
| VmchB_R |  | GAGCAAGTAAGTGTCCTGAC |
| dmchCD_F | Delete *mchC* and *mchD* | AACACTCTTTTATGAAAATTGAAACGGAGAATACTGCCAGGCTGGAGCTGCTTC |
| dmchCD_R |  | TACCCCGTAATTTACAATTATCTCTGTATCTTCAGAGGTACATATGAATATCCTCCTTA |
| VmchCD_F | Verify *mchC mchD* deletion, transpose *mchC* *mchD* for complementation | AGTGGTAGTGCCAGTTCTTC |
| VmchCD_R |  | TGCTTCCCAGCATGATTAAC |
| dmchEF_F | Delete *mchE* and *mchF* | AGCTATTTTGTTTCGTCAGGATGCTTTAGAAAACAGAAAAGCTGGAGCTGCTTC |
| dmchEF_R |  | CCATCGACAAAACTCCTTACAAAGTTACACCAGTGGATTTCATATGAATATCCTCCTTA |
| VmchEF_F | Verify *mchE, mchF* deletion, transpose *mchE, mchF* for complementation | CGCAGATCTCCTCTGAAGATACAGAGAT |
| VmchEF_R |  | CCCAAGCTTCCATAGTAATAACTAAGAT |
| JPN42 (11) | Verify and transpose *clbA* deletion | CAGATACACAGATACCATTCA |
| JPN46 (11) |  | CTAGATTATCCGTGGCGATTC |
| dPCPclbH_F | Delete ClbH peptidyl carrier protein (PCP) domain | GGCCGGGCGAGTATGTCCCTCCGGCAACCGAACTGGAACAGCGTCTGGCGGCTGGAGCTGCTTC |
| dPCPclbH_R |  | ACGTGCTGACCGCACTGCTGCCCGATGTATTGCTGCATCTGTACTAGCAGCATATGAATATCCTCCTTAG |
| VPCPclbH_F | Verify deletion of ClbH PCP domain | GCAGTGGTTAACGGTTATGG |
| VPCPclbH_R |  | GGAATCAGTGATGACTGTCG |
| IHAPJPN61 (20) | Verify and transpose *clbL* deletion | CAACTGCACAATCTACCCGCC |
| IHAPJPN62 (20) |  | GCTGTCACCGATATCCGCCTC |
| dclbM_F | Delete *clbM* | CCATCGGCATGCAGCGGTTGGATCGTGCACAGCGGATTTTGTCGGTCGGTATCATCATCGCGCTATTCTT |
| dclbM_R |  | CAATGTGTAGATGAAATAGGCGACCATACTCAGTTGCGCCAGAAACGACGCCAATGCCACCCCTTTCACA |
| AS60 | Verify *clbM* deletion | GGTGACAGCGCCAATAAAG |
| AS140 |  | GATGTGGCACTGTTCCTG |
| IHAPJPN29 (20) | Verify and transpose *clbP* deletion | GTGAACTGAGCGAAATATTGGCTAATC |
| IHAPJPN30 (20) |  | TTACTCATCGTCCCACTCCTTGTTG |
| PB-clbP_pASK75_F | Amplify ClbP signal sequence with overlapping primers for pASK and PhoA | CGGTACCCGGGGATCCATGACAATAATGG |
| PB-clbPps_phoA_R |  | CAGGCATCGCCCCGATAGGCTCATG |
| PB-phoA_clbPps_F | Amplify the enzymatic domain of the alkaline phosphatase PhoA with primers overlapping ClbP signal sequence and C-terminal sequence | CCTATCGGGGCGATGCCTGTTCTGGAAAACC |
| PB-phoA_clbP3H_R |  | CGCAACGCAATTTTCAGCCCCAGAGCGGC |
| PB-clbP3H_phoA_F | Amplify the 3 C-terminal transmembrane helices of ClbP with primers overlapping PhoA and pASK75 sequences | GGGCTGAAAATTGCGTTGCGACCGGATCAG |
| PB-clbP3H_pASK75_R |  | GTCGACCTCGAGTTACTCATCGTCCCACTCC |
| PB-pASK75_clbP3H_F | Amplify pASK75 sequence with primers overlapping ClbP signal sequence and C-terminal sequence | GATGAGTAACTCGAGGTCGACCTGCAGGCAG |
| PB-pASK75_clbPps_R |  | CCATTATTGTCATGGATCCCCGGGTACCG |
| IRSDNG26 | Insert the S95A mutation in the *clbP* gene sequence | CCTGAATCAGTATTTGCACCACAAGTCCGGTAAACGCCTTACTCATCcgTCCCAGCTCGTAAACTGTGTCTAGAGTATTCGCTTTCTGAC |
| IHAPJPN55 (20) | Verify and transpose *clbQ* deletion | TTATCCTGTTAGCTTTCGTTC |
| IHAPJPN56 (20) |  | CTTGTATAGTTACACAACTATTTC |
| entD-P1 (11) | Delete *entD* | GGGCGGATCGCTGCAATTTATGCGCTGCGGGAATATGGCTATAAATGTGTGCgtgtaggctggagctgcttc |
| entD-P2 (11) |  | TCACTTGCCTTAAATGCGCTCTCTTTGGCGGAAAATGCCAGTGTCAGCGCcatatgaatatcctccttag |
| entE-P1 (11) | Delete *entE* | TATCGACGGCGAGCGACAGTTGAGTTATCGGGAGCTGAATCAGGCGGCgtgtaggctggagctgcttc |
| entE-P2 (11) |  | AAGCGCAGCTTTTTTCGCCCATCAGCTCATCTTCCATGCTCACCAGTGCcatatgaatatcctccttag |
| diroB_F | Delete *iroB* | CATGACTCCTGGATGGGGGCTGGCAAATGATTATCATGAAGGTGTAGTGGGCTGGAGCTGCTTC |
| diroB_R |  | GAGTGCGTCGACTGCCTGATTTAGATCGTCAAGCGGAGAGGGATTTTCTCCATATGAATATCCTCCTTAG |
| ViroB_F | Verify *iroB* deletion | CCACGATGACACTAAGTTTC |
| ViroB_R |  | AAAGTCGTGTCCTGCTGCTG |
| IRSD NG7 | Transpose *iroB* into pBbA5a-RFP | GCTTTAGAATTCTTTAGATCGTCAAGCGGAGAGG |
| IRSD NG8 |  | CTTTAGGATCCCTACCCTTTCTGTACCATTGTG |
| diroC_F | Delete *iroC* | ACGGTACCGCCGCTTCTGACTGTGTTTTTAATACATTACGTGTCCAGTAAGCTGGAGCTGCTTC |
| diroC_R |  | GGCGAAAAGCCTGATCACAATGGTACAGAAAGGGTAGCCACTACACCTTCCATATGAATATCCTCCTTAG |
| ViroC_F | Verify *iroC* deletion | CCAGAAGTCAGTAAGGTTAGC |
| ViroC_R |  | GGTGAAGTGGCGAAAAG |
| diroD_F | Delete *iroD* | GATTTATTCAGGAAGTCTGTACCGTTTCCTGAAAGTGTTTGTGGGTCAACGCTGGAGCTGCTTC |
| diroD_R |  | TAGCCGCTTTAATTATTTATACTGACATCCTTAATTTTTAAAGAGTATGACATATGAATATCCTCCTTAG |
| ViroD_F | Verify *iroD* deletion | CAGGAAGTCTGTACCGTTTCC |
| ViroD_R |  | ATCGCCAGCACAACATGAAG |
| diroE_F | Delete *iroE* | TTAGTGGCTTAACTCATGACAACCTGCTGTGTAATTTGCGTTTTCACCACTGCATATGAATATCCTCCTTAG |
| diroE_R |  | ATGTATGCCCGCGAGTATCGCTCAACACGCCCGCATAAAGCGATTTTCTTTCCGTGTAGGCTGGAGCTGCTTC |
| ViroE_F | Verify *iroE* deletion | GAGTTACCGCATCATTCTGA |
| ViroE_R |  | TTGTCACCTGCGGAAAAGG |
