# Supplementary material for: Deciphering the interplay between the genotoxic and probiotic activities of Escherichia coli Nissle 1917
Source: PLoS Pathog. 2019 Sep 23;15(9):e1008029. doi: 10.1371/journal.ppat.1008029 (PMC6776366; doi:10.1371/journal.ppat.1008029)
Supplement: S3 Table — (DOCX) [file ppat.1008029.s003.docx]

**Table S3. Genbank accession numbers for genome sequences included in Figure 7B**

| Strain | Accession number |
| --- | --- |
| *E. coli* str. 042 | FN554766.1 |
| *E. coli* str. 317 | [CP023357.1](https://www-ncbi-nlm-nih-gov.gate2.inist.fr/nucleotide/CP023357.1?report=genbank&log$=nucltop&blast_rank=8&RID=9ZE2GP3H014) |
| *E. coli* str. 536 | [CP000247.1](https://www-ncbi-nlm-nih-gov.gate2.inist.fr/nucleotide/CP000247.1?report=genbank&log$=nucltop&blast_rank=24&RID=9ZCFRBUV014) |
| *E. coli* str. 1105 | [CP023388.1](https://www-ncbi-nlm-nih-gov.gate2.inist.fr/nucleotide/CP023388.1?report=genbank&log$=nucltop&blast_rank=3&RID=9ZCK4WYW015) |
| *E. coli* str. ABU83972 | [CP001671.1](https://www-ncbi-nlm-nih-gov.gate2.inist.fr/nucleotide/CP001671.1?report=genbank&log$=nucltop&blast_rank=24&RID=9ZCK4WYW015) |
| *E. coli* str. ACN002 | [CP007491.1](https://www-ncbi-nlm-nih-gov.gate2.inist.fr/nucleotide/CP007491.1?report=genbank&log$=nucltop&blast_rank=31&RID=9ZCK4WYW015) |
| *E. coli* str. ATCC^®^25922 | CP009072.1 |
| *E. coli* str. BH100 MG2017 | CP025251.1 |
| *E. coli* str. CFT073 | [AE014075.1](https://www-ncbi-nlm-nih-gov.gate2.inist.fr/nucleotide/AE014075.1?report=genbank&log$=nucltop&blast_rank=25&RID=9ZCK4WYW015) |
| *E. coli* clone D i2 | [CP002211.1](https://www-ncbi-nlm-nih-gov.gate2.inist.fr/nucleotide/CP002211.1?report=genbank&log$=nucltop&blast_rank=23&RID=9ZCK4WYW015) |
| *E. coli* clone D i14 | [CP002212.1](https://www-ncbi-nlm-nih-gov.gate2.inist.fr/nucleotide/CP002212.1?report=genbank&log$=nucltop&blast_rank=22&RID=9ZCK4WYW015) |
| *E. coli* str. D8 | [CP010151.1](https://www-ncbi-nlm-nih-gov.gate2.inist.fr/nucleotide/CP010151.1?report=genbank&log$=nucltop&blast_rank=7&RID=9ZCK4WYW015) |
| *E. coli* str. ECONIH2 | [CP014667.1](https://www-ncbi-nlm-nih-gov.gate2.inist.fr/nucleotide/CP014667.1?report=genbank&log$=nucltop&blast_rank=23&RID=9ZCFRBUV014) |
| *E. coli* str. FORC_028 | [CP012693.1](https://www-ncbi-nlm-nih-gov.gate2.inist.fr/nucleotide/CP012693.1?report=genbank&log$=nucltop&blast_rank=30&RID=9ZCK4WYW015) |
| *E. coli* str. FORC_029 | [CP013185.1](https://www-ncbi-nlm-nih-gov.gate2.inist.fr/nucleotide/CP013185.1?report=genbank&log$=nucltop&blast_rank=8&RID=9ZCK4WYW015) |
| *E. coli* str. FORC_043 | [CP016828.1](https://www-ncbi-nlm-nih-gov.gate2.inist.fr/nucleotide/CP016828.1?report=genbank&log$=nucltop&blast_rank=5&RID=9ZCK4WYW015) |
| *E. coli* str. IHE3034 | [CP001969.1](https://www-ncbi-nlm-nih-gov.gate2.inist.fr/nucleotide/CP001969.1?report=genbank&log$=nucltop&blast_rank=28&RID=9ZE2GP3H014) |
| *E. coli* str. K-15KW01 | [CP016358.1](https://www-ncbi-nlm-nih-gov.gate2.inist.fr/nucleotide/CP016358.1?report=genbank&log$=nucltop&blast_rank=22&RID=9ZCFRBUV014) |
| *E. coli* str. LF82 | CU651637.1 |
| *E. coli* str. MRY15-117 | [AP017617.1](https://www-ncbi-nlm-nih-gov.gate2.inist.fr/nucleotide/AP017617.1?report=genbank&log$=nucltop&blast_rank=13&RID=9ZCFRBUV014) |
| *E. coli* str. NCCP15648 | [CP009050.1](https://www-ncbi-nlm-nih-gov.gate2.inist.fr/nucleotide/CP009050.1?report=genbank&log$=nucltop&blast_rank=4&RID=9ZCK4WYW015) |
| *E. coli* str. Nissle 1917 | [CP007799.1](https://www-ncbi-nlm-nih-gov.gate2.inist.fr/nucleotide/CP007799.1?report=genbank&log$=nucltop&blast_rank=17&RID=9ZCK4WYW015) |
| *E. coli* str. NU14 | CP019777.1 |
| *E. coli* str. O104:H4 str. C227-11 | [CP011331.1](https://www-ncbi-nlm-nih-gov.gate2.inist.fr/nucleotide/CP011331.1?report=genbank&log$=nucltop&blast_rank=10&RID=9ZCK4WYW015) |
| *E. coli* str. UPEC 26-1 | [CP016497.1](https://www-ncbi-nlm-nih-gov.gate2.inist.fr/nucleotide/CP016497.1?report=genbank&log$=nucltop&blast_rank=9&RID=9ZCK4WYW015) |
| *E. coli* str. UTI89 | [CP000243.1](https://www-ncbi-nlm-nih-gov.gate2.inist.fr/nucleotide/CP000243.1?report=genbank&log$=nucltop&blast_rank=31&RID=9ZE2GP3H014) |
