# Supplementary figures and images for: Deciphering the interplay between the genotoxic and probiotic activities of Escherichia coli Nissle 1917
Source: PLoS Pathog. 2019 Sep 23;15(9):e1008029. doi: 10.1371/journal.ppat.1008029 (PMC6776366; doi:10.1371/journal.ppat.1008029)

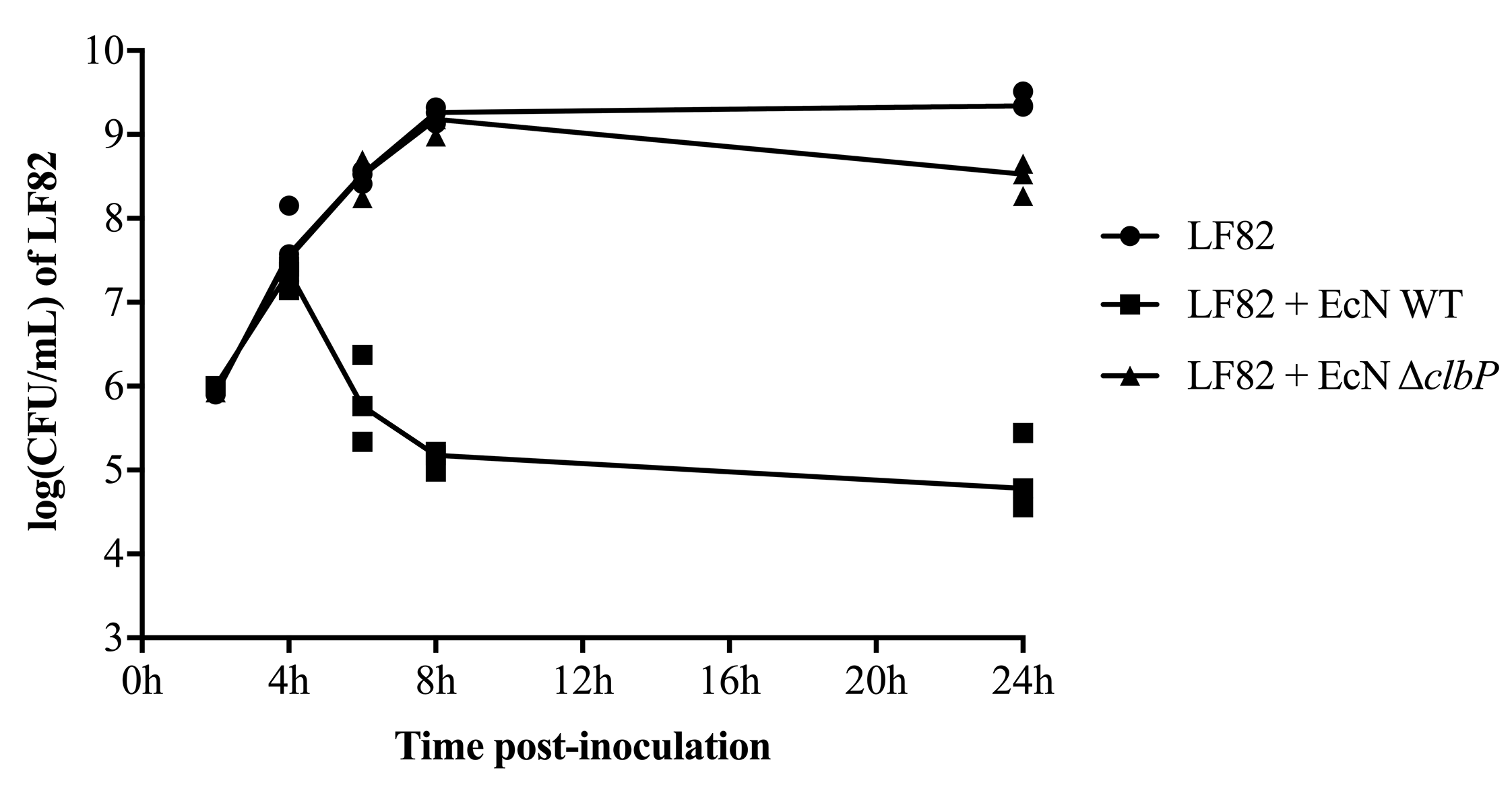

Supplement: S1 Fig — Colony forming unit (CFU) counts of E. coli LF82 following a 2, 4, 6, 8, and 24-hour co-culture in M63 medium with the wild-type (WT) E. coli strain Nissle 1917 (EcN) or the mutant for colibactin maturing peptidase ClbP (ΔclbP). LF82 was also cultured alone as a control. The individual results of the 3 independent experiments are shown and the medians for each time point are linked. (TIF) [file ppat.1008029.s004.tif]

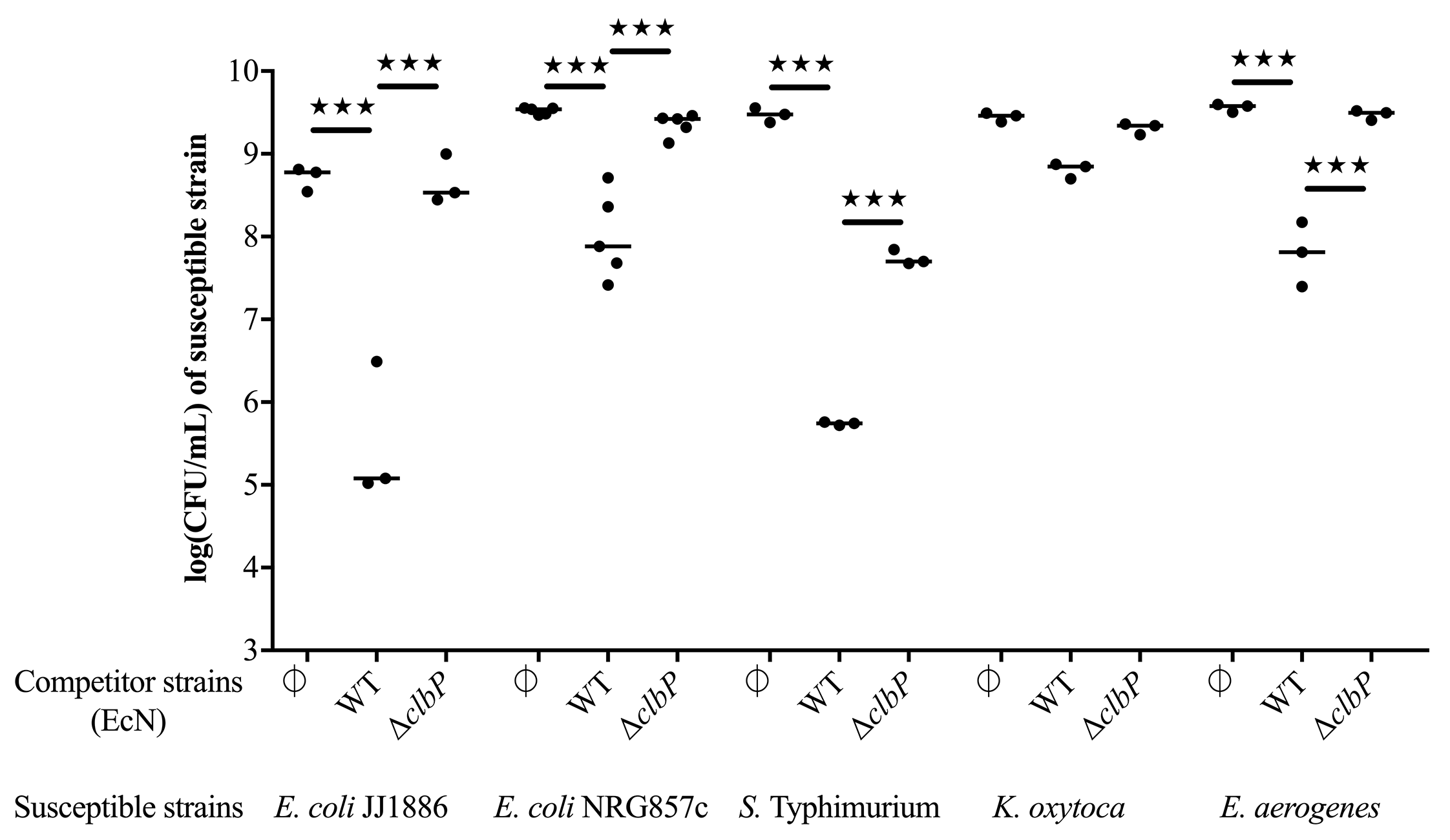

Supplement: S2 Fig — Colony forming unit (CFU) counts of E. coli ST131 isolate JJ1886, E. coli NRG857c, Salmonella enterica serovar Typhimurium IR715, Enterobacter aerogenes ATCC®13048, and Klebsiella oxytoca ATCC®13182 after a 24-hour co-culture in Dulbecco’s Modified Eagle Medium with wild-type (WT) E. coli strain Nissle (EcN), and EcN ΔclbP mutant. All susceptible strains were also cultured alone as controls (∅). The medians and individual results of independent experiments are shown. One-way ANOVA and Bonferroni post-tests; ★★★P<0.001. (TIF) [file ppat.1008029.s005.tif]

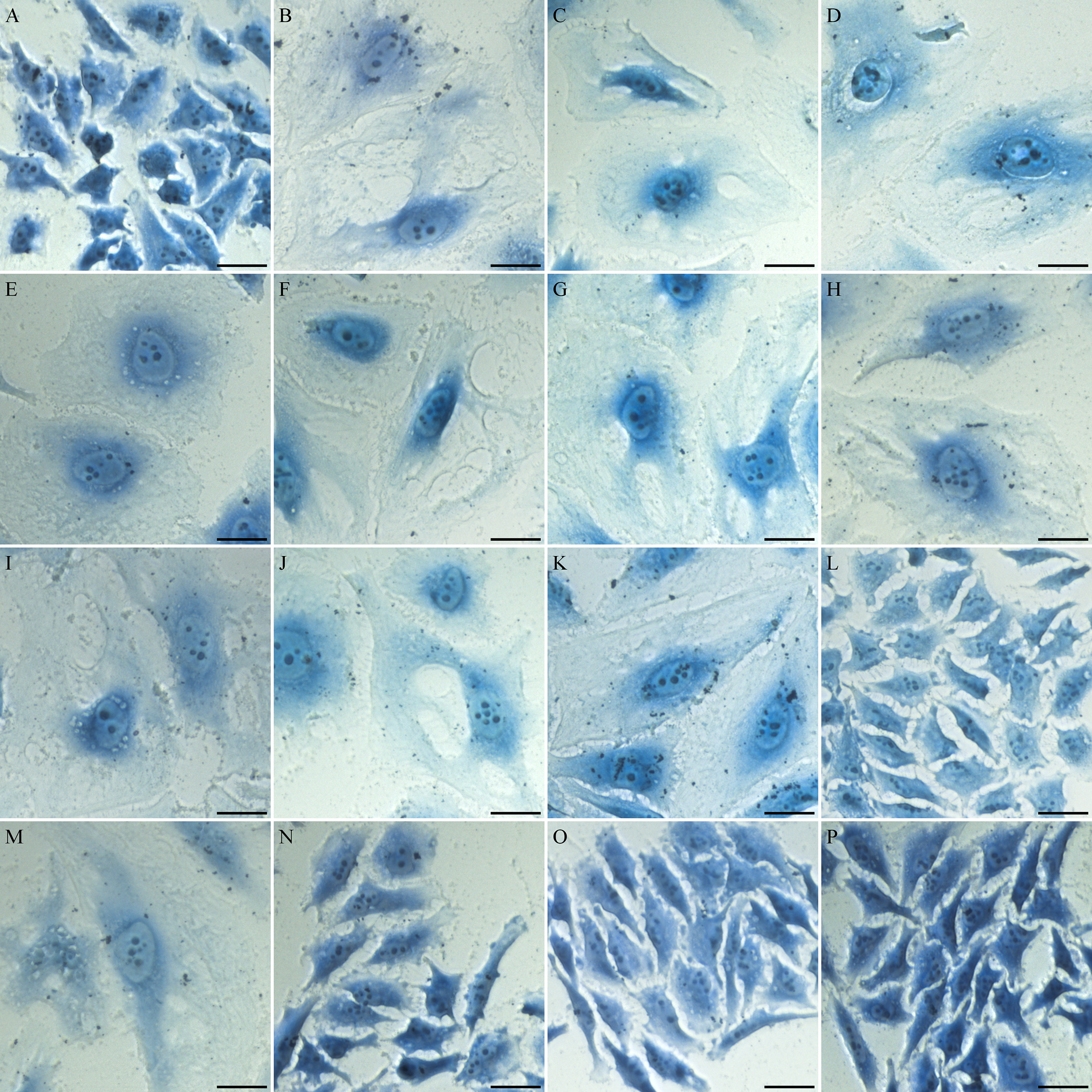

Supplement: S3 Fig — HeLa cells were transiently infected with wild-type (WT) E. coli strain Nissle (EcN) (B) and various mutants then washed and incubated with gentamicin for 72 hours before staining with Giemsa. The control is shownd in A. Bars represent 50 μm. Mutations in the EcN microcin gene cluster did not abrogated EcN cytopathic activity: EcN mutants for C. mcmA; D. mchB; E. mcmAmchB; F. mchCD; G. mchEF. Mutations in the EcN salmochelin gene cluster did not abrogated EcN cytopathic activity: EcN mutants for H. iroB; I. iroC; J. iroD; K. iroE. EcN ΔclbP mutant (L) did not affect cells, whereas clbP complementation restored the cytopathic activity (M). Complementation of EcN ΔclbP mutant with a plasmid encoding ClbP with an S95A mutation (N), a K98T mutation (O) did not restore the cytopathic activity, as well as complementation with the plasmid encoding the fusion between alkaline phosphatase and the C-terminal domain of ClbP (P). (TIF) [file ppat.1008029.s006.tif]

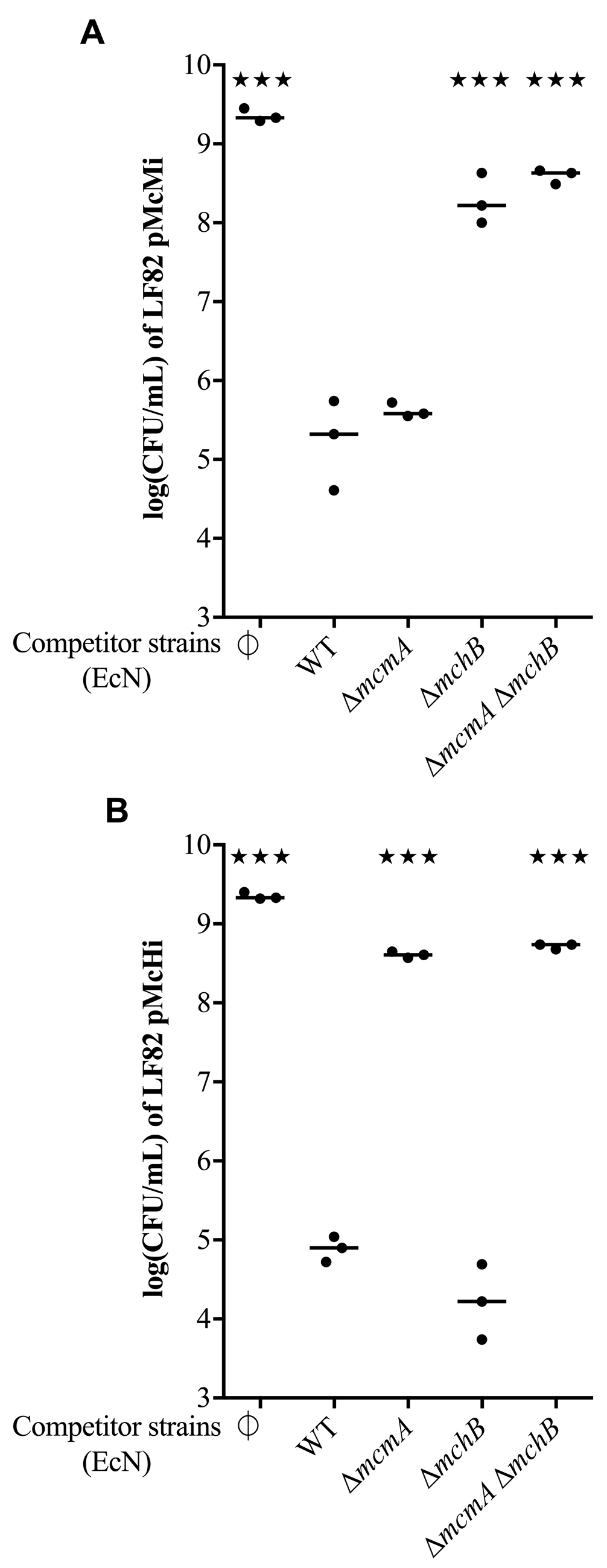

Supplement: S4 Fig — Colony forming unit (CFU) counts of E. coli LF82 carrying; A: a plasmid encoding microcin M (MccM) immunity gene mcmI (pMcMi); B: microcin H47 (MccH47) immunity gene mchI (pMcHi) after a 24-hour co-culture in M63 minimal medium with wild-type (WT) E. coli strain Nissle (EcN), EcN mutants for MccM precursor gene mcmA, for MccH47 precursor gene mchB, and for both mcmA mchB genes. LF82 carrying pMcMi or pMcHi were also cultured alone as controls (∅). The medians and individual results of independent experiments are shown. One-way ANOVA and Bonferroni post-tests in comparison with co-culture with WT; ★★★P<0.001. (TIF) [file ppat.1008029.s007.tif]

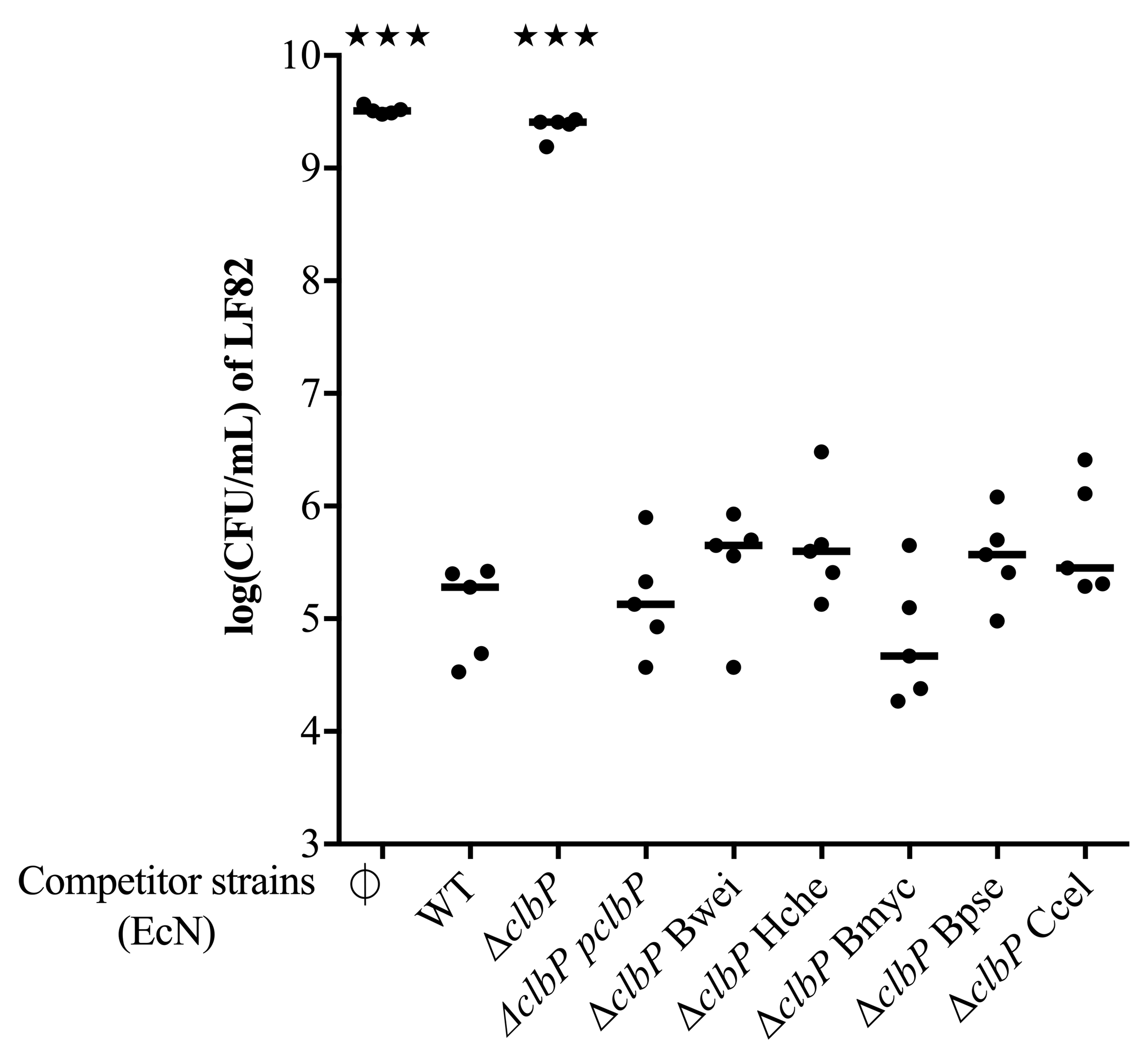

Supplement: S5 Fig — Colony forming unit (CFU) counts of E. coli LF82 following a 24-hour-coculture in M63 medium with wild-type (WT) E. coli strain Nissle 1917 (EcN), the clbP mutant and complemented strain with a plasmid encoding wild-type ClbP (pclbP), with plasmids encoding ClbP-like proteins from Bacillus weihenstephanensis (Bwei), Hahella chejuensis (Hche), B. mycoides (Bmyc), B. pseudomycoides (Bpse), and Clostridium cellulolyticum (Ccel). LF82 was also cultured alone as a control (∅). Medians and individual results of independent experiments are shown. One-way ANOVA and Bonferroni post-tests in comparison with coculture with WT; ★★★P<0.001. (TIF) [file ppat.1008029.s008.tif]

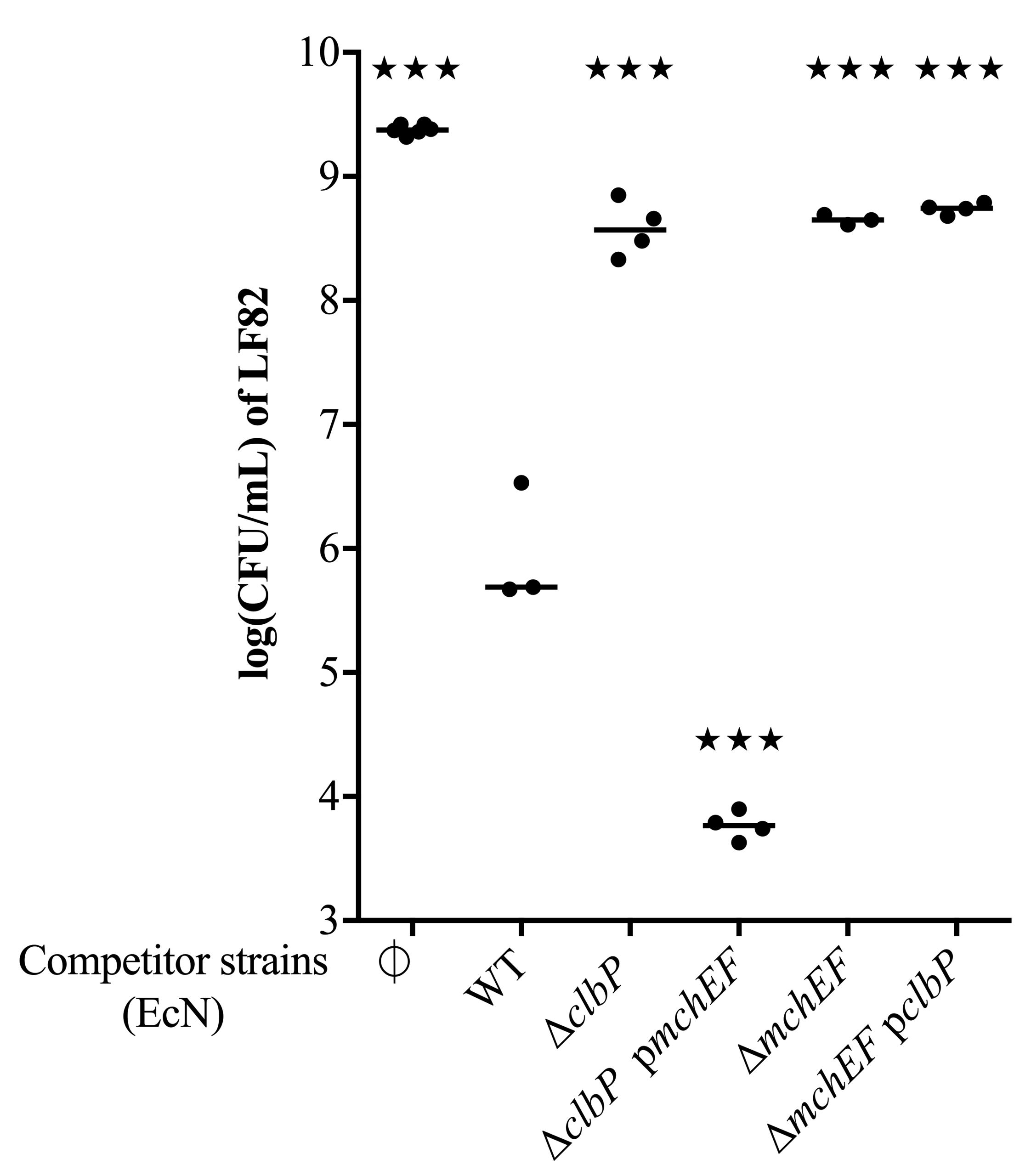

Supplement: S6 Fig — Colony forming unit (CFU) counts of E. coli LF82 following a 24-hour co-culture in M63 medium with WT EcN, EcN mutant for the peptidase ClbP and the corresponding mutant overexpressing the efflux pump MchEF (pmchEF), EcN mutant for MchEF and the corresponding mutant overexpressing ClbP (pclbP). LF82 was also cultured alone as a control (∅). The medians and individual results of independent experiments are shown. One-way ANOVA and Bonferroni post-tests in comparison with co-culture with WT; ★★★P<0.001. (TIF) [file ppat.1008029.s009.tif]

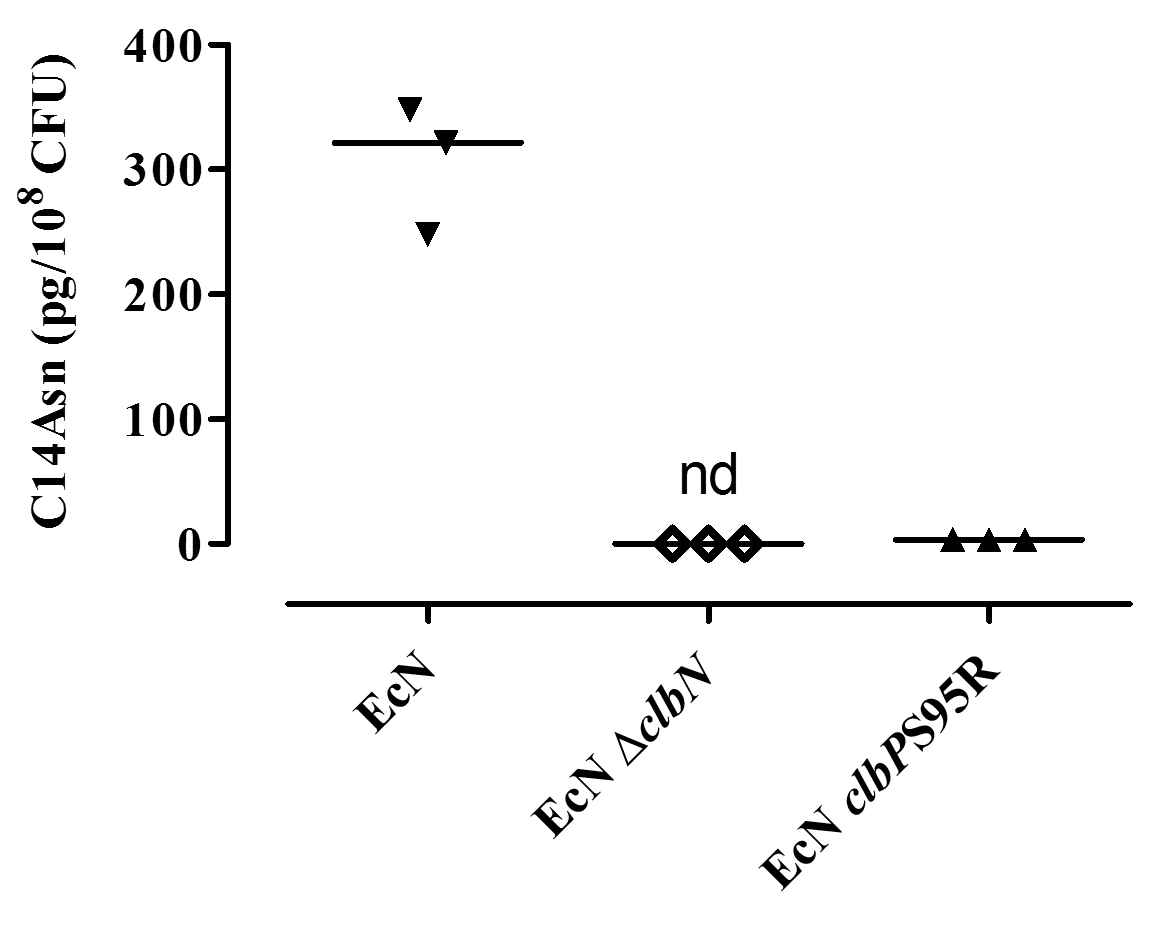

Supplement: S7 Fig — E. coli Nissle (EcN), a clbN deletion mutant and the genome edited clbP-S95R mutant were grown 24 h in DMEM in triplicate cultures, then the bacteria were pelleted, the lipids were extracted and the colibactin cleavage product C14-Asn was quantified by liquid chromatography coupled to mass spectrometry. The results of the triplicate tubes and median are shown. Nd: none detected. (TIF) [file ppat.1008029.s010.tif]

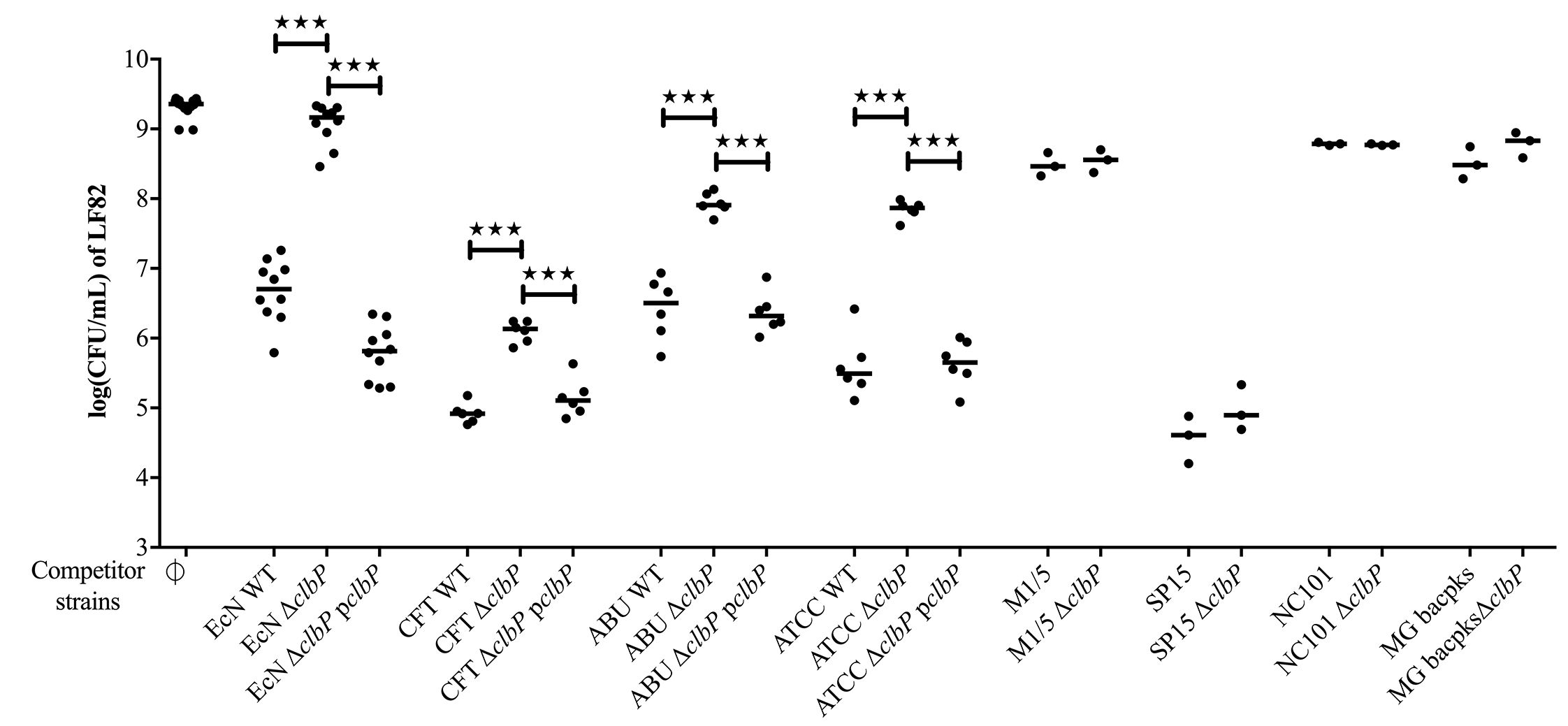

Supplement: S8 Fig — Colony forming unit (CFU) counts of E. coli LF82 following a 24-hour co-culture in Dulbecco’s Modified Eagle Medium with E. coli strains that carry both a truncated microcin gene cluster and the pks island: wild-type (WT) E. coli strains Nissle 1917 (EcN), CFT073 (CFT), ABU83972 (ABU), ATCC 25922 (ATCC), the corresponding clbP gene deletion mutants and complemented mutant strains, and pks+ E. coli strain M1/5 (human commensal), SP15 (responsible for meningitis), NC101 (murine commensal and procarcinogenic), MG1655 that hosts a bacterial artificial chromosome (BAC) bearing the pks island (MGbacpks), and the corresponding clbP gene deletion mutants. LF82 was also cultured alone as a control (∅). Medians and individual results of independent experiments are shown. One-way ANOVA and Bonferroni post-tests; ★★★P<0.001. (TIF) [file ppat.1008029.s011.tif]

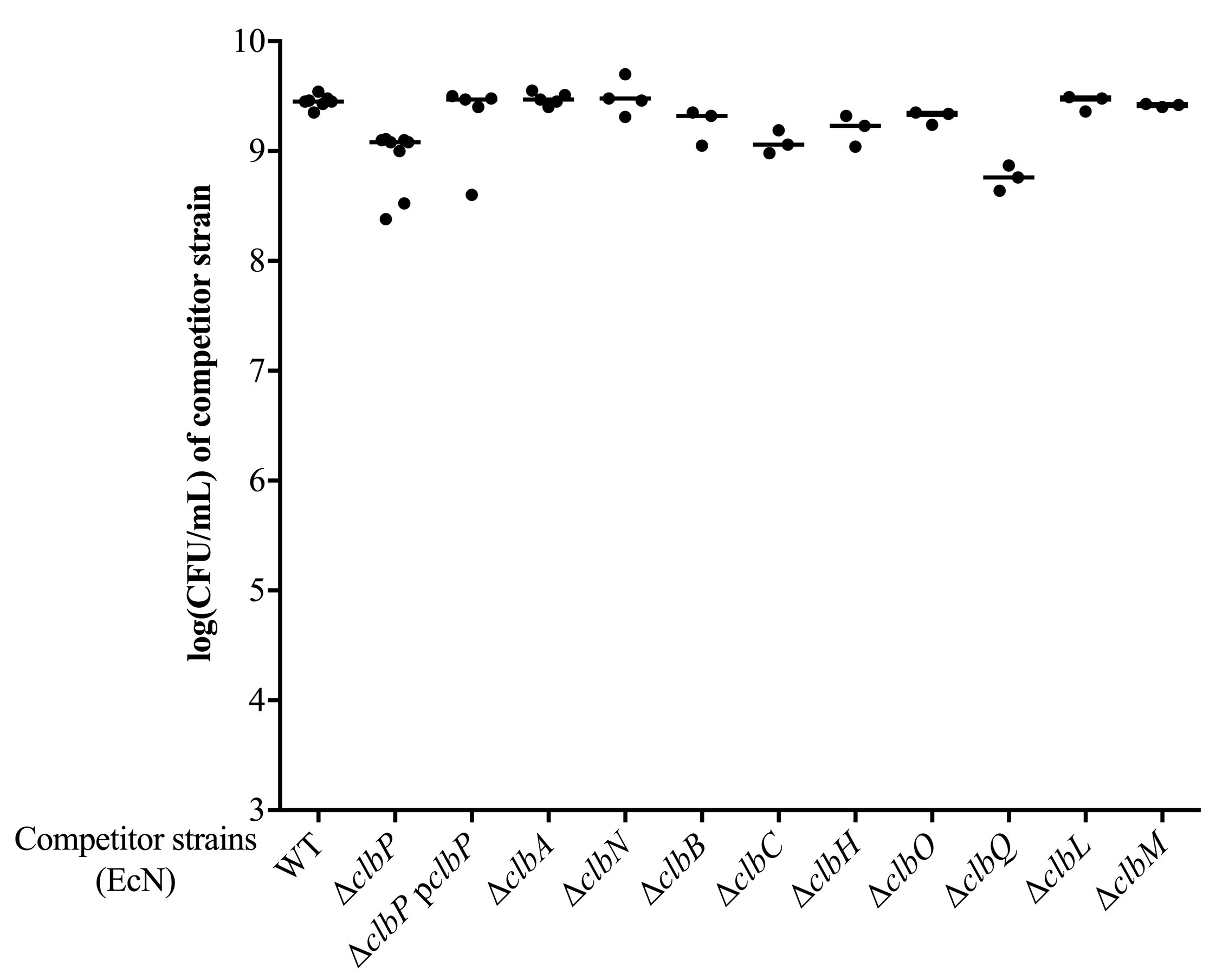

Supplement: S9 Fig — Colony forming unit (CFU) counts of EcN and its mutants following a 24-hour co-culture in Dulbecco’s Modified Eagle Medium with E. coli LF82. (TIF) [file ppat.1008029.s012.tif]
